# MINocyclinE to Reduce inflammation and blood‐brain barrier leakage in small Vessel diseAse (MINERVA): A phase II, randomized, double‐blind, placebo‐controlled experimental medicine trial

**DOI:** 10.1002/alz.13830

**Published:** 2024-04-17

**Authors:** Robin B. Brown, Daniel J. Tozer, Laurence Loubière, Eric L. Harshfield, Young T. Hong, Tim D. Fryer, Guy B. Williams, Martin J. Graves, Franklin I. Aigbirhio, John T. O'Brien, Hugh S. Markus

**Affiliations:** ^1^ Department of Clinical Neurosciences University of Cambridge Cambridge UK; ^2^ Wolfson Brain Imaging Centre University of Cambridge Cambridge UK; ^3^ Department of Radiology University of Cambridge Cambridge Cambridge UK; ^4^ Department of Psychiatry University of Cambridge Cambridge UK

**Keywords:** blood‐brain barrier, cerebral small vessel disease, neuroinflammation, vascular cognitive impairment, vascular dementia

## Abstract

**INTRODUCTION:**

Cerebral small vessel disease (SVD) is a common cause of stroke/vascular dementia with few effective treatments. Neuroinflammation and increased blood‐brain barrier (BBB) permeability may influence pathogenesis. In rodent models, minocycline reduced inflammation/BBB permeability. We determined whether minocycline had a similar effect in patients with SVD.

**METHODS:**

MINERVA was a single‐center, phase II, randomized, double‐blind, placebo‐controlled trial. Forty‐four participants with moderate‐to‐severe SVD took minocycline or placebo for 3 months. Co‐primary outcomes were microglial signal (determined using ^11^C‐PK11195 positron emission tomography) and BBB permeability (using dynamic contrast‐enhanced MRI).

**RESULTS:**

Forty‐four participants were recruited between September 2019 and June 2022. Minocycline had no effect on ^11^C‐PK11195 binding (relative risk [RR] 1.01, 95% confidence interval [CI] 0.98–1.04), or BBB permeability (RR 0.97, 95% CI 0.91–1.03). Serum inflammatory markers were not affected.

**DISCUSSION:**

^11^C‐PK11195 binding and increased BBB permeability are present in SVD; minocycline did not reduce either process. Whether these pathophysiological mechanisms are disease‐causing remains unclear.

**INTERNATIONAL CLINICAL TRIALS REGISTRY PORTAL IDENTIFIER:**

ISRCTN15483452

**Highlights:**

- We found focal areas of increased microglial signal and increased blood‐brain barrier permeability in patients with small vessel disease.
- Minocycline treatment was not associated with a change in these processes measured using advanced neuroimaging.
- Blood‐brain barrier permeability was dynamic but MRI‐derived measurements correlated well with CSF/serum albumin ratio.
- Advanced neuroimaging is a feasible outcome measure for mechanistic clinical trials.

## 1 BACKGROUND

Cerebral small vessel disease (SVD) accounts for around 25% of strokes, and is the most common pathology underlying vascular cognitive impairment.^1^ Despite its public health significance, the underlying disease mechanisms are not completely understood and effective disease‐modifying treatments are limited.^2^

SVD typically occurs in patients with cardiovascular risk factors such as hypertension, hypercholesterolaemia, smoking, and diabetes.^3^ However, treatment of these risk factors has a limited effect in slowing disease progression, and therefore alternative pathophysiological mechanisms have been investigated to guide further therapeutic intervention.

One such process is inflammation, both systemic and within the central nervous system (CNS). Inflammatory cells, particularly microglia, have been found in the white matter in post‐mortem patients with SVD,^4^ and elevation of cerebrospinal fluid (CSF) biomarkers such as matrix metalloproteases MMP‐2, MMP‐9, and TIMP‐1 has been reported.^5^

Evidence for CNS inflammation is also provided by positron emission tomography (PET), using radioligands such as ^11^C‐PK11195 targeted against the translocator protein (TSPO), a mitochondrial surface protein upregulated in microglial activation. Both increased global ^11^C‐PK11195 binding, and small foci or “hotspots” of increased binding, have been reported in SVD.^6^ Elevated blood biomarkers of inflammation have also been reported.^7^, ^8^

Another recently implicated process is increased permeability of the blood‐brain barrier (BBB).^9^ Immunoglobulin and fibrinogen deposition have been shown in *post mortem* brains from patients with SVD,^10^ consistent with BBB leakage at some time. An increased CSF/serum albumin ratio, a marker of BBB permeability, has been reported in both lacunar stroke and subcortical vascular dementia,^5^, ^11^.

Increased BBB permeability can also be measured non‐invasively, using dynamic contrast‐enhanced MRI (DCE‐MRI) to measure the T_1_ relaxation time change after administration of a gadolinium based contrast agent (GBCA). Increased permeability occurs in SVD, not only in white matter lesions but also in “normal appearing white matter” (NAWM), and is correlated with radiological SVD severity.^12^ Advanced image analysis allows spatial mapping of BBB permeability,^11^ and “hotspots” of increased BBB permeability have been shown in the white matter in SVD.^6^

If inflammation and increased BBB permeability do play a causal role in SVD pathogenesis, then targeting them might represent a novel treatment approach. This hypothesis was examined in a rodent model of white matter ischemia.^13^ Increases in hypoxia inducible factor‐1α were seen, followed by infiltrating T cells and neutrophils, with matrix metalloproteinase‐9 (MMP‐9) co‐localizing with the inflammatory cells, followed by BBB leakage. Minocycline, which has anti‐inflammatory actions including MMP‐9 inhibition, reduced white matter lesion volume and improved behavioral outcomes.

This suggests that targeting neuroinflammation and BBB leakage in man might represent a novel therapeutic approach for SVD. We tested whether the results of the rodent study could be replicated in a double‐blind phase 2 randomized controlled trial, with advanced imaging outcome measures of neuroinflammation (^11^C‐PK11195 PET) and BBB leakage (DCE‐MRI) measured simultaneously using a PET/MRI scanner.

## 2 METHODS

### 2.1 Study design

The MINocyclinE to Reduce inflammation and blood brain barrier leakage in small Vessel diseAse (MINERVA) study was a phase II randomized, double‐blind placebo‐controlled trial that recruited 44 participants with symptomatic SVD from a single center in Cambridge, UK between September 2019 and June 2022.

### 2.2 Participants

Participants were eligible if they were 18 years or older and had symptoms consistent with SVD (either a clinical lacunar stroke with an anatomically corresponding lacunar infarct, gait apraxia, or self‐reported cognitive impairment), and moderate or higher white matter hyperintensity (WMH) burden on MRI scan (Fazekas score ≥ 2). Exclusion criteria included any cause of stroke other than SVD, cortical infarcts, a subcortical infarct >1.5 cm diameter, monogenic forms of SVD, cerebral amyloid angiopathy, contraindications to MRI or minocycline, dementia diagnosis, woman of childbearing potential, or glomerular filtration rate of ≤59 mL/min/m^2^ in view of gadolinium administration. The trial protocol has been published.^14^ All participants were studied at least 3 months after last stroke to reduce the impact of acute inflammatory changes. Figure S1 shows detailed inclusion and exclusion criteria.

### 2.3 Randomization and masking

Randomization was performed using a Web‐based system (www.sealedenvelope.com). Participants were randomly allocated to receive active treatment or placebo in a 1:1 ratio using a randomly permuted block randomization with block sizes of two and four. All participants, study personnel, and investigators were blinded to treatment allocation. One author (L.L.) generated the allocation sequence, and another (R.B.B.) randomized the participants and allocated them to treatment.

### 2.4 Procedures

After undergoing an initial screening visit and providing written, informed consent, participants were randomized to receive minocycline 100 mg twice daily (or matching placebo) for 3 months. The baseline visit included phlebotomy, neuropsychometric testing and PET/MRI imaging, and they were then provided with bottles containing either over‐encapsulated minocycline tablets or matching cellulose‐filled placebo capsule.

**FIGURE 1:**
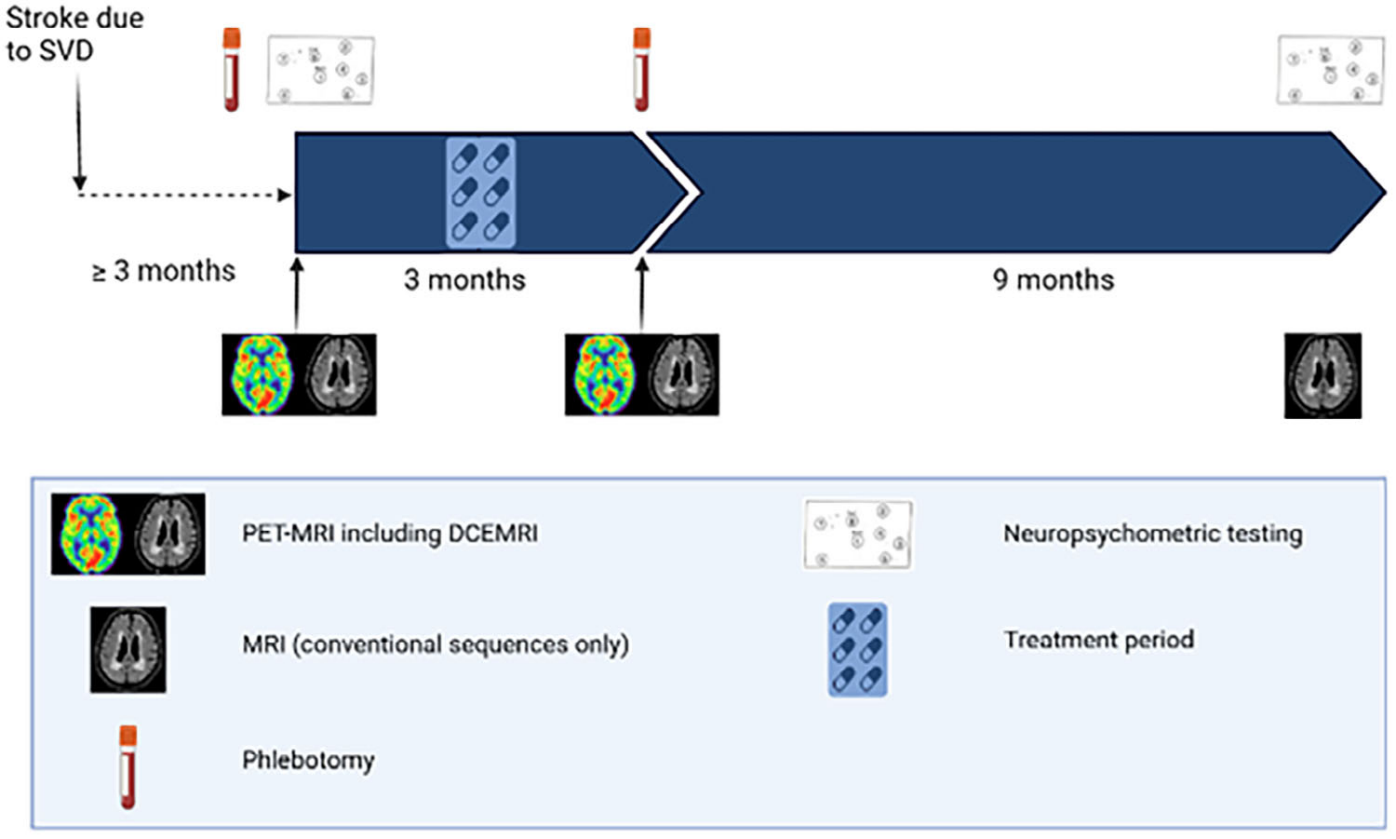
Structured study diagram showing timeline and trial procedures.

Participants attended at 6 weeks for a clinical check‐up; 3 months (13 weeks) for further data collection, phlebotomy, and repeat PET/MRI imaging; and at 1‐year for non‐contrast MRI and repeat neuropsychometric testing. Trial design is summarized in Figure 1.

**RESEARCH IN CONTEXT**

1. **Systematic review**: The authors reviewed existing literature using PubMed, Google Scholar, abstracts from meetings and relevant references from these sources. There is considerable evidence that inflammation and blood‐brain barrier permeability play a role in small vessel disease pathophysiology. These relevant citations are appropriately cited.
2. **Interpretation**: Our results do not suggest a role for minocycline as a disease‐modifying therapy. Mechanistic trials in small vessel disease are feasible using outcomes based on advanced imaging.
3. **Future directions**: Longitudinal follow‐up data from the MINERVA trial will allow us to test the hypothesis that minocycline has beneficial effects over a longer term period based on clinical and radiological outcomes. We will also be able to test whether microglial signal and blood‐brain barrier permeability at baseline predicts the risk of cognitive decline.

### 2.5 Outcomes

1. Primary co‐endpoints:
  a) The percentage volume of ^11^C‐PK11195 binding “hotspots” in normal appearing white matter (NAWM) measured on PET.
  b) Percentage volume of BBB permeability “hotspots” in NAWM measured on MRI.
2. Secondary endpoints:
  a) Mean ^11^C‐PK11195 binding potential and mean BBB permeability in NAWM.
  b) Mean binding potential of hotspots of ^11^C‐PK11195 and mean gadolinium transfer constant of hotspots of BBB permeability in NAWM.
  c) Blood endothelial and inflammatory markers.

### 2.6 Imaging acquisition

PET and MRI were simultaneously co‐acquired on a 3T GE SIGNA PET/MRI scanner (GE Healthcare, Waukesha, WI, USA) at the Wolfson Brain Imaging Centre in Cambridge, UK using sequences previously optimized in a similar cohort.^6^ Full details of the imaging acquisition have been published.^14^ Baseline and 3‐month (post‐treatment) imaging comprised:

1. PET data acquisition for 75 min following the injection of ^11^C‐PK11195 (target injection activity 500 MBq), produced at the Wolfson Brain Imaging Centre Radiopharmaceutical Unit, Cambridge, UK.
2. Simultaneous whole brain MRI using a 32‐channel head coil (Nova Medical) including T_1_‐ and T_2_‐weighted images, FLAIR, DTI, and susceptibility weighted images. Full sequence details are in Table S1.
3. Dynamic quantitative T_1_ maps acquired using DCE‐MRI in a sub‐volume of the brain chosen to reflect characteristic SVD lesions. Gadoterate meglumine, a GBCA (Dotarem), was injected at a sub‐clinical dose of 0.025 mmol/kg. The T_1_ relaxation time was mapped prior to injection, and then followed by eight cycles of post‐contrast T_1_ mapping; each T_1_ map was calculated using an in‐house developed pulse sequence that acquires six 3D radiofrequency (RF) spoiled gradient echo image sets with different flip angles.

### 2.7 Image analysis

Image analysis pipelines have previously been published^6^, ^14^ and are summarized here. WMH lesions were delineated using a semi‐automated contouring program (Jim version 8.0; http://xinapse.com/j‐im‐software/), with baseline and follow‐up images marked slice by slice on a parallel split screen and the rater blinded to image timepoint.^15^ T_1_‐weighted images were processed using SIENAX from the FSL package (https://fsl.fmrib.ox.ac.uk/fsl/fslwiki/) to produce tissue probability maps for each tissue class after removal of the WMH mask. WMHs and NAWM masks were subsequently eroded by 3 mm to eliminate contamination from CSF or gray matter.

Quantitative T_1_ maps from the DCE‐MRI were calculated using the standard RF spoiled‐gradient echo signal equation. Patlak graphical analysis was then applied to estimate gadolinium concentration in tissue and to determine influx rate (*K_i_*) as a metric of permeability.^16^ As the field of view does not include an artery, the superior sagittal sinus was used as an arterial input function, corrected by the factor (1‐hematocrit), which is assumed to be representative of arterial input.^11^ Voxels of increased BBB permeability (“hotspots”) were defined as those with *K_i_* greater than the 95th percentile of permeability derived from an existing cohort of stroke‐free control participants, scanned using the same protocol and hardware.

List‐mode PET data were histogrammed into 55 discrete time bins and reconstructed into images using time‐of‐flight ordered subsets expectation‐maximization,^17^ with 16 subsets, six iterations, and no smoothing. Attenuation correction included the use of a multi‐subject atlas method^18^ and improvements to the MRI brain coil component.^19^ Image reconstruction corrected for random coincidences, dead time, normalization, scattered coincidences, radioactive decay, and sensitivity. SPM12 was used to realign each dynamic image series which was then co‐registered with the T_1_ MRI sequence using a mean realigned PET image.

The binding potential (BP_ND_) of ^11^C‐PK11195 relative to a non‐displaceable reference tissue (BP_ND_), a metric of binding site density, was estimated using a basis function implementation of the simplified reference tissue model that incorporates correction for vascular binding.^20^ The white matter reference tissue input was estimated by supervised cluster analysis,^21^ using library data from ^11^C‐PK11195 scans in healthy control participants using the same PET‐MRI scanner. BP_ND_ hotpots were defined as those above the 95th percentile of control participants as for BBB permeability measurements above.

### 2.8 Blood sampling and processing

10 mL blood was collected in the morning before each imaging appointment. After being left for at least 30 min to clot, the samples were centrifuged at 1000 × *g* for 15 min stored at −80°C for en bloc analysis. High sensitivity C‐reactive protein (hsCRP) was measured at the University of Cambridge Core Biochemical Assay Laboratory using an enzyme‐linked immunosorbent assay (Siemens Healthineers, Erlangen, Germany). A sample was sent for proteomic analysis using the Olink Cardiovascular III platform, a panel of 92 protein biomarkers related to cardiovascular disease and inflammation, including cell adhesion molecules such as ICAM‐2; matrix metalloproteases such as MMP‐3, MMP‐9, and TIMP‐4; and conventional makers of cardiovascular endothelial activation or angiogenesis including vWF and t‐PA (Olink, Sweden; https://www.olink.com/products‐services/target/cardiometabolic‐panel/).

### 2.9 Cerebrospinal fluid sampling

In a subgroup of participants who consented additionally, we performed lumbar puncture at baseline. The cerebrospinal fluid (CSF)/serum albumin ratio was calculated to confirm validity of the DCE‐MRI BBB measurements (see Supplementary Material for methods).

### 2.10 Safety and adverse event reporting

At each trial visit, data were collected on potential side effects. Additional safety outcomes included recurrent stroke or other cardiovascular events.

### 2.11 Data capture/data access

Data were recorded electronically using an online research data management tool (Research Electronic Data Capture, REDCap).^22^ An interim database lock was performed on February 22, 2023, after the final participant completed treatment and all entries up to the 3‐month follow‐up were quality controlled.

### 2.12 Protocol adjustments and deviations due to COVID‐19 pandemic

Several amendments were made to trial procedures in response to coronavirus disease 2019 (COVID‐19). No in‐person research visits were permitted between March and July 2020, and the Research Ethics Committee approved extending treatment for participants who had baseline visits and were on treatment prior to the pandemic, up to a maximum of 6 months, to allow them to have follow‐up scans and blood tests while still on treatment. This affected two participants.

Four participants were randomized before the pandemic but were not able to participate when research visits reopened in July 2020 (two due to medical issues and two due to concerns about attending the site during the ongoing pandemic), and were therefore replaced to achieve the sample size of 44. As no data were collected beyond screening information, these four participants were not included in any analysis.

During UK lockdown periods in 2020/2021 we did not require participants’ renal function to be checked prior to the appointment; this was considered unjustifiable exposure. Instead, we checked it on the day itself and did not perform DCE‐MRI if renal function was below 60 mL/min/1.73 m^2^, leading to four participants having imaging conventional MRI and PET only without the DCE‐MRI.

### 2.13 Statistical analysis

Sample size calculations were based on data from our observational study.^6^ We calculated to show a 20% reduction in ^11^C‐PK11195 BP_ND_ binding metrics with power of 80% and *α* = 0.05, we would require 17 participants in each arm, and 21 to demonstrate a 20% reduction in BBB *K_i_*. To account for dropouts, we chose a sample size of 44.

We performed the primary outcome analysis on an intention‐to‐treat (ITT) basis. We also performed a per‐protocol analysis, including only participants who completed the treatment course.

We compared differences in demographic and vascular risk factors between the treatment groups, using two‐sample *t*‐tests to compare continuous traits, χ2 tests to compare binary traits, or Fisher's exact test if frequency was less than five for any group, and quantile regression of medians to compare ordinal traits.

We tested association of treatment group with each imaging outcome using linear regression models both unadjusted and adjusted for age, in both ITT and per‐protocol populations. We analyzed associations of treatment with each individual blood biomarker. We also performed a principal component analysis (PCA) on the biomarkers from the proteomic panel and analyzed associations of treatment group with each of the first three principal components.

All statistical analyses were conducted using *R* version 4.3.0 (R Core Team, 2023). Results are presented using two‐sided *p*‐values and 95% confidence intervals. We applied Bonferroni correction to account for multiple testing comparisons when identifying significant associations of each individual blood protein with each outcome measure.

### 2.14 Ethical and regulatory approval

Approval for the MINERVA trial was granted by the East of England, Cambridge Central Research Ethics Committee (reference 18/EE/0237). The use of ^11^C‐PK11195 was approved by the UK Administration of Radioactive Substances Advisory Committee (ARSAC, Research ID 176; September 19, 2018). The study was registered on the International Clinical Trials Registry Portal (reference ISRCTN15483452).

## 3 RESULTS

### 3.1 Participant characteristics

**FIGURE 2:**
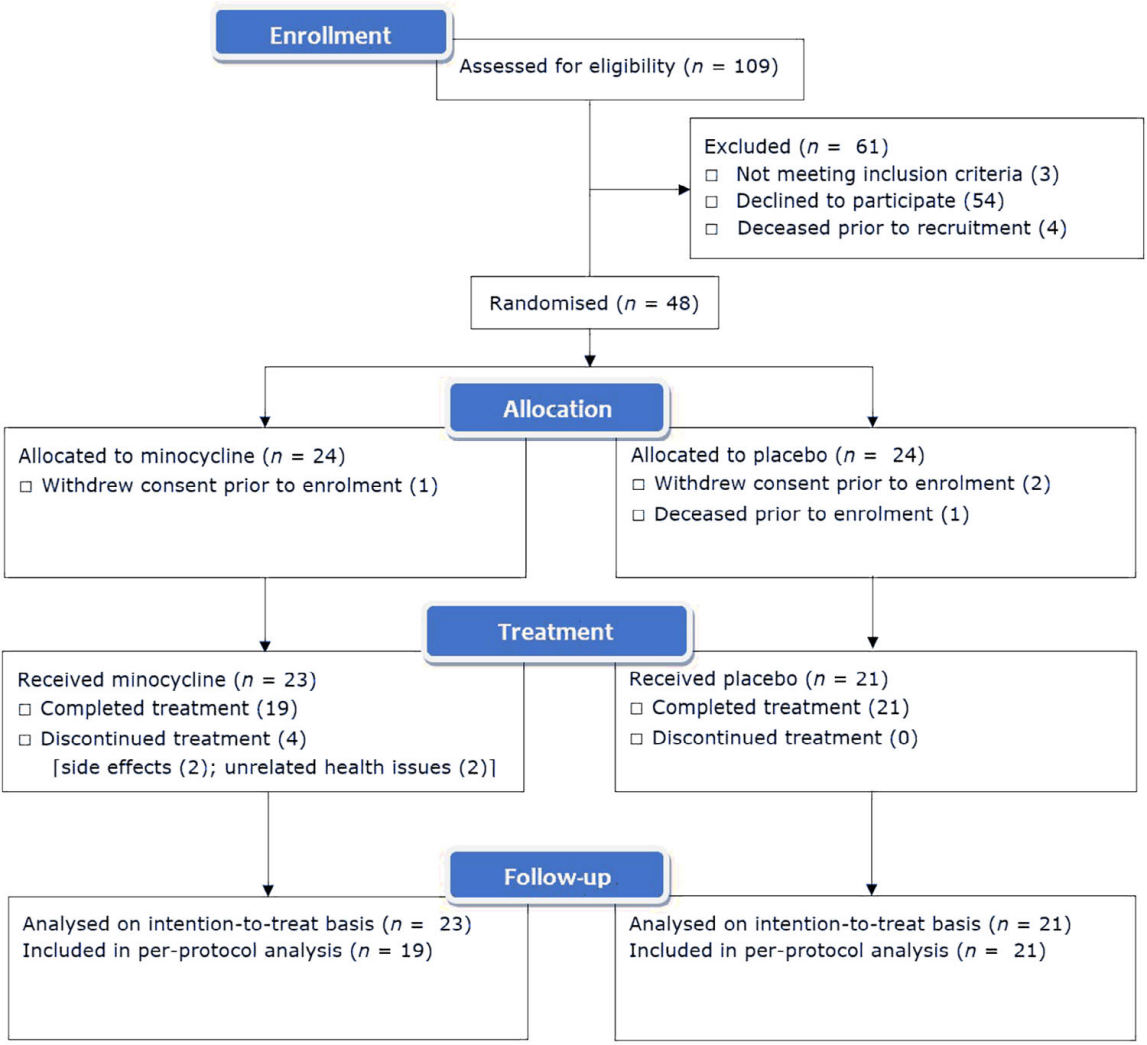
CONSORT recruitment diagram showing flow of participants in the MINERVA study. MINERVA, minocycline to reduce inflammation and blood brain barrier leakage in small vessel disease.

**FIGURE 3 alz13830-fig-0003:**
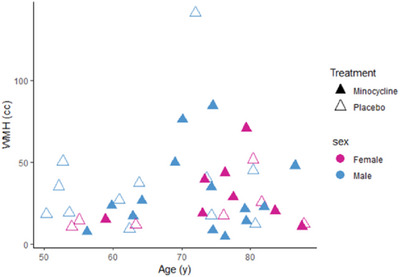
Graphical representation of intention‐to‐treat cohort stratified by age, sex, and disease severity (WMH volume). WHM, white matter hyperintensity.

109 participants were screened for eligibility between September 2019 and June 2022, of whom 61 were excluded (Figure 2). Due to the COVID‐19 pandemic (see the Methods section), 4 randomized patients did not enter the study and therefore, 48 participants were randomized (minocycline 24; placebo 24) to achieve the sample size of 44 (minocycline 23; placebo 21) Presenting symptoms were lacunar stroke in 43, and cognitive impairment in 1. Mean age of 69.9 ± 10.8 years, and 28/44(63.6%) were male. Mean WMH volume was 31.3 ± 26.0 cc, indicating moderate to severe white matter disease. Table 1 shows the participant characteristics. Participants in the intervention group were older with borderline statistical significance (mean age 73.0 ± 8.9 vs. 66.5 ± 11.9 years, *p *= 0.04); accordingly, we present analysis of primary and secondary outcomes both unadjusted and corrected for age. There were no other between‐group differences in demographics, comorbidities, or radiological markers of SVD severity (Table 1). Figure 3 shows the trial cohort stratified by age, WMH volume, sex, and treatment allocation.

**TABLE 1:** Between group comparisons for the ITT population showing a borderline significant difference in age only.

	Minocycline	Placebo	Overall	
Parameter	(*N* = 23)	(*N* = 21)	(*N* = 44)	*p‐*Value
Age (years)	73.0 (8.90)	66.5 (11.9)	69.9 (10.8)	0.04
Sex				
Female	9 (39.1%)	7 (33.3%)	16 (36.4%)	0.69
Male	14 (60.9%)	14 (66.7%)	28 (63.6%)	
Ethnicity—White British				
Yes	20 (87.0%)	19 (90.5%)	39 (88.6%)	1.00
No	3 (13.0%)	2 (9.5%)	5 (11.4%)	
Education (years)	12 [12–14]	14 [12–17]	13 [12–14]	0.14
Hypertension				
Yes	22 (95.7%)	16 (76.2%)	38 (86.4%)	0.09
No	1 (4.4%)	5 (23.8%)	6 (13.6%)	
Hyperlipidemia				
Yes	15 (65.2%)	18 (85.7%)	33 (75.0%)	0.17
No	8 (34.8%)	3 (14.3%)	11 (25.0%)	
Ischemic heart disease				
Yes	3 (13.0%)	1 (4.8%)	4 (9.1%)	0.61
No	20 (87.0%)	20 (95.2%)	40 (90.9%)	
Diabetes mellitus				
Yes	3 (13.0%)	5 (23.8%)	8 (18.2%)	0.45
No	20 (87.0%)	16 (76.2%)	36 (81.8%)	
Body mass index (kg/m^2^)	30.9 (10.1)	30.2 (7.5)	30.5 (8.8)	0.80
Current smoker				
Yes	4 (17.4%)	2 (9.5%)	6 (13.6%)	0.67
No	19 (82.6%)	19 (90.5%)	38 (86.4%)	
Time since stroke (months)	24.8 (28.3)	21.1 (20.4)	23.1 (24.7)	0.64
WMH (cc)	31.2 (22.6)	31.5 (30.0)	31.3 (26.0)	0.98
Lacunes	2 [1–3]	2 [1–3]	2 [1–3]	1.00
CMBs	0 [0–3]	0 [0–2]	0 [0–2]	1.00
Brain volume (cc)	1420.5 (82.4)	1426.8 (69.2)	1423.4 (75.7)	0.79
Mean ^11^C‐PK11195 injected activity (baseline; MBq)	393.1 (88.2)	443.4 (75.1)	415.7 (85.4)	0.06
Mean ^11^C‐PK11195 injected activity (follow‐up; MBq)	416.1 (57.7)	433.4 (44.9)	424.2 (52.0)	0.33

*Note*: Values are mean (SD) or median [IQR]. *p*‐Value is from *t*‐test, χ**2** test (or Fisher's exact test if frequency is less than five for any comparison), or quantile regression of medians, as appropriate.

Abbreviations: ITT, intention‐to‐treat; WMH, white matter hyperintensity.

Thirty‐eight participants completed the MRI protocol pre‐ and post‐treatment, including 32 who had DCE‐MRI at both timepoints. Thirty participants completed PET imaging pre‐ and post‐treatment, and serum biomarker results were acquired for 43/44 (Table 2). No participants had a confirmed infection with COVID‐19 or suffered a typical upper respiratory tract illness during the treatment period.

**TABLE 2 alz13830-tbl-0002:** Number of participants who completed conventional MRI imaging, DCE‐MRI imaging, PET imaging, and blood tests both pre‐ and post‐treatment.

		Minocycline	Placebo	Overall
Treatment time	Parameter	(*N* = 23)	(*N* = 21)	(*N* = 44)
**Baseline**	MRI	22	19	41
	DCE‐MRI	17	16	33
	PET	20	15	35
	Blood	22	21	43
**Follow‐up**	MRI	20	18	38
	DCE‐MRI	16	16	32
	PET	17	13	30
	Blood	22	21	43

Abbreviations: DCE‐MRI, dynamic contrast‐enhanced magnetic resonance imaging; PET, positron emission tomography.

### 3.2 Primary outcomes

**FIGURE 4 alz13830-fig-0004:**
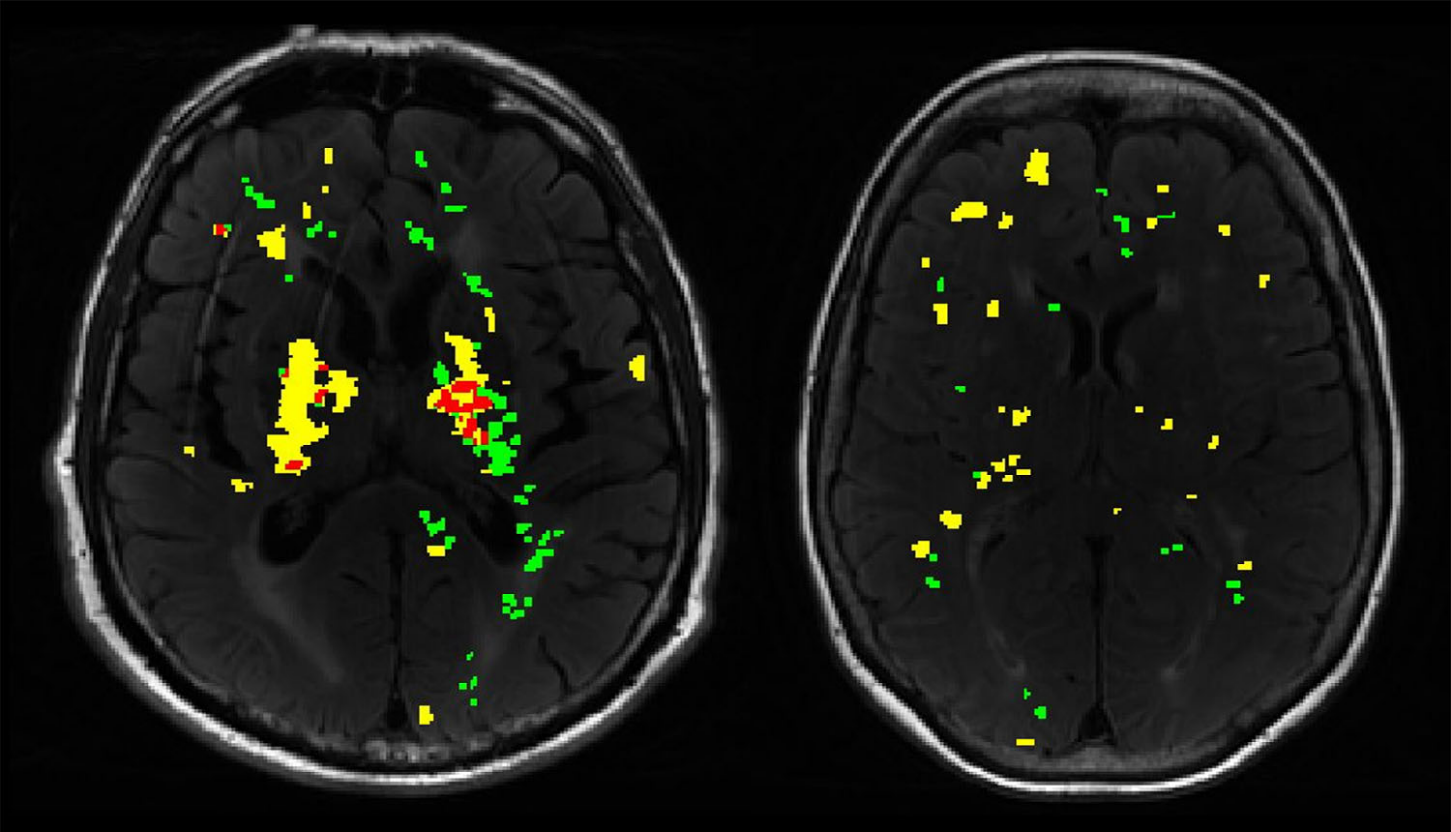
Representative images from two patients showing maps of hotspots of ^11^C‐PK11195 binding (green) and BBB permeability (yellow) overlaid on FLAIR images. Overlapping voxels defined as hotposts of both processes are colored red (only present in left panel). BBB, blood‐brain barrier; FLAIR, fluid attenuated inversion recovery.

Hotspots of both ^11^C‐PK11195 binding and increased BBB permeability were seen in patients. Representative images are shown in Figure 4.

#### 3.2.1 ^11^C‐PK11195 binding hotspots

**FIGURE 5 alz13830-fig-0005:**
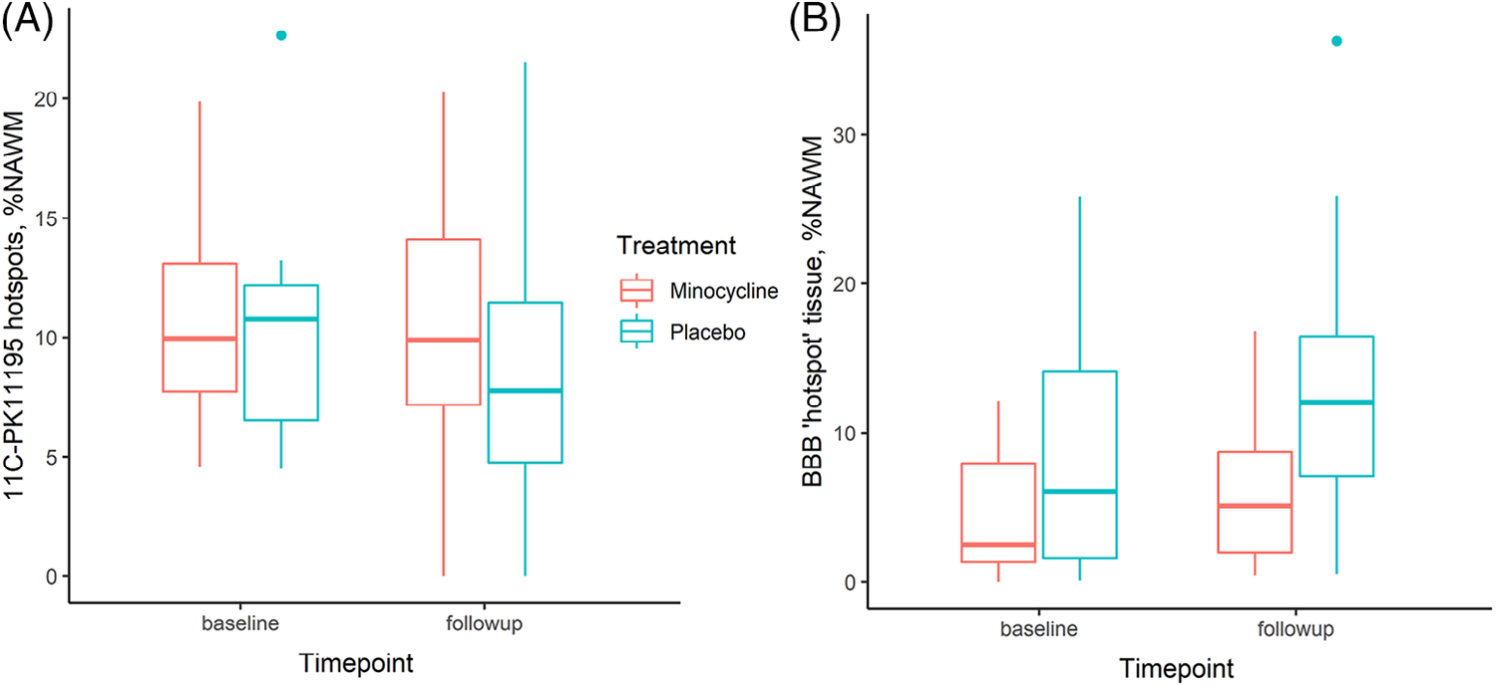
Boxplots showing between‐group comparison pre‐ and post‐treatment for (A) mean percentage of NAWM identified as ^11^C‐PK11195 binding ‘hotspot’ tissue volume (percentage of NAWM) and (B) BBB permeability hotspot volume (percentage of NAWM). Boxes show group mean/quartiles, whiskers show maximum values within 1.5 * inter‐quartile range of upper or lower quartile, outliers beyond these values are plotted separately. BBB, blood‐brain barrier; NAWM, normal appearing white matter.

The mean percentage of NAWM identified as ^11^C‐PK11195 binding “hotspot” tissue at baseline did not differ between groups (minocycline 10.71 ± 4.04%, placebo 10.11 ± 4.67%, *p *= 0.69). At follow‐up, the percentage hotspot tissue was 9.97 ± 5.50% on minocycline and 7.79 ± 5.67% in the placebo group. On ITT analysis, there was no treatment effect of minocycline on the change in percentage of hotspots (relative risk [RR] 1.01, 95% CI 0.98–1.04; see Table 3); correction for age made no difference (RR 1.01, 95% CI 0.97–1.05). Group average values pre‐ and post‐treatment are shown in Figure 5A.

**TABLE 3 alz13830-tbl-0003:** Effect of treatment on change in volume of BBB permeability hotspots, volume of ^11^C‐PK11195 binding hotspots, mean BBB transfer constant (*K_i_*) and ^11^C‐PK11195 BP_ND_ in NAWM, and hotspot mean *K_i_* and ^11^C‐PK11195 BP_ND_ in ITT population.

Parameter	RR (95% CI)	*p*‐Value
**Primary analyses**		
Change in BBB permeability hotspot volume in eroded NAWM	0.97 (0.91–1.03)	0.27
Adjusted for age	0.97 (0.91–1.04)	0.41
Change in ^11^C‐PK11195 BP_ND_ hotspot volume in eroded NAWM	1.01 (0.98–1.04)	0.58
Adjusted for age	1.01 (0.97–1.05)	0.62
**Secondary analyses**		
Change in mean *K_i_* in all eroded NAWM	1.00 (1.00–1.00)	0.70
Adjusted for age	1.00 (1.00–1.00)	0.83
Change in mean ^11^C‐PK11195 BP_ND_ in all eroded NAWM	0.99 (0.98–1.01)	0.23
Adjusted for age	0.99 (0.98–1.00)	0.17
Change in mean *K_i_* of BBB permeability hotspot in eroded NAWM	1.00 (0.99–1.01)	0.35
Adjusted for age	1.00 (0.99–1.01)	0.39
Change in mean BP_ND_ of ^11^C‐PK11195 binding hotspot in eroded NAWM	1.00 (0.96–1.06)	0.85
Adjusted for age	1.01 (0.96–1.07)	0.60

Abbreviations: BBB, blood‐brain barrier; BPND, binding potential; ITT, intention‐to‐treat; NAWM, normal appearing white matter.

#### 3.2.2 BBB permeability hotspots

The mean percentage of NAWM identified as BBB permeability “hotspot” tissue at baseline did not differ between groups (minocycline 4.08 ± 3.69%, placebo 8.49 ± 8.45%, *p *= 0.07). At follow‐up, percentage of hotspot tissue was 6.19 ± 5.09% on[Fig alz13830-fig-0003], [Table alz13830-tbl-0002], [Fig alz13830-fig-0004] minocycline and 13.04 ± 9.24% in the placebo group. On ITT analysis, there was no treatment effect of minocycline on change in percentage of hotspots (RR 0.97, 95% CI 0.91–1.03; see Table 3). This result was unaltered after co‐varying for age (RR 0.97, 95% CI 0.91–1.04). Group average values pre‐ and post‐treatment are shown in Figure 5B.

### 3.3 Secondary outcomes

#### 3.3.1 White matter ^11^C‐PK11195 binding potential

^11^C‐PK11195 BP_ND_ in NAWM was highly correlated between timepoints (Pearson's *r* 0.785, 95% CI 0.608‐0.887; see Figure S2 and Table S2). Mean ^11^C‐PK11195 BP_ND_ at baseline was −0.036 ± 0.025 in the treatment group and −0.041 ± 0.024 mL/g/min in the placebo group. At follow‐up, mean ^11^C‐PK11195 BP_ND_ was −0.032 ± 0.032 in the treatment group and −0.028 ± 0.027 in the placebo group. On ITT analysis, there was no treatment effect (RR 0.99, 95% CI 0.98–1.01); adjustment for age did not affect the results.

Mean ^11^C‐PK11195 BP_ND_ of hotspot tissue was 0.189 ± 0.018 in the treatment group and 0.183 ± 0.019 in the placebo group. At follow‐up, the mean BP_ND_ of hotspot tissue was 0.150 ± 0.076 in the treatment group and 0.131 ± 0.094 in the placebo group). On ITT analysis, there was no treatment effect of minocycline on the change in hotspot mean BP_ND_ either unadjusted or adjusted for age.

#### 3.3.2 White matter BBB permeability

Mean BBB transfer constant (*K_i_*) values in the NAWM were poorly correlated between baseline and follow‐up scans (Pearson's *r* −0.089, 95% CI −0.430–0.274; see Figure S3 and Table S3). Mean *K_i_* at baseline was 0.0002 ± 0.0003 mL/g/min in the treatment and 0.0008 ± 0.001 mL/g/min in the placebo group. At follow‐up, mean *K_i_* was 0.0002 ± 0.0003 mL/g/min in the treatment and 0.0005 ± 0.0004 mL/g/min in the placebo group. On ITT analysis, there was no treatment effect (RR 1.00, 95% CI 1.00–1.00); results were similar adjusted for age.

Mean *K_i_* of hotspot tissue was 0.007 ± 0.009 mL/g/min in the treatment and 0.011 ± 0.015 mL/g/min in the placebo group. At follow‐up, mean *K_i_* of hotspot tissue was 0.004 ± 0.002 mL/g/min in the treatment and 0.004 ± 0.002 mL/g/min in the placebo group. There was no treatment effect on the change in hotspot means *K_i_* either unadjusted or adjusted for age.

#### 3.3.3 Serum biomarkers

There was no treatment effect on hsCRP or on any of the individual biomarkers in the Olink CVD III panel when corrected for multiple comparisons. Minocycline was not associated with change in serum levels in three MMPs involved in ECM remodeling and implicated in the rodent model (MMP‐2, RR 1.18, 95% CI 1.01–1.38, not significant when corrected for multiple comparisons; MMP‐3, RR 0.97, 95% CI 0.73–1.29; and MMP‐9, RR 1.60, 95%CI 0.97–2.65). After performing PCA on the biomarkers, the first three principal components explained >42% of the variance. There were no treatment effects on any of the first three principal components, either unadjusted or adjusted for age. Data for individual biomarkers and PCA components are presented in Tables S4–S6 and Figure S4.

#### 3.3.4 Additional outcome measurements

As a sensitivity analysis, we tested whether the neuroimaging outcomes were altered if the white matter was not eroded (to check for any bias that may have arisen from significant volumes of tissues being removed in participants with higher WMH volume). There was no material change to the results, with no treatment effect identified on any of the primary or secondary outcomes (Table S7).

We also performed a per‐protocol analysis including only those participants who completed treatment, which showed no effect of minocycline on the primary outcomes or any of the secondary outcomes (including serum biomarkers; Tables S8–S11 and Figure S5).

### 3.4 Safety and adverse events

11/23 participants[Table alz13830-tbl-0003] (47.8%) in the minocycline group and 7/21 participants (33.3%) in the placebo group had side effects (*p *= 0.59). Two participants stopped treatment due to side effects, both in the minocycline group. Table S12 shows the adverse effect profile stratified by treatment group.

Two serious adverse events were reported, both in minocycline participants. One stopped treatment due to acute pancreatitis, which was adjudicated to be unrelated to trial medication, and one had a recurrent stroke 1 week after stopping trial treatment.

### 3.5 Validation of DCE‐MRI BBB permeability measurements

In the subgroup of 12 participants who consented to additional lumbar puncture at baseline, the mean CSF/serum albumin ratio was 5.39 ± 1.71 mg/g. The CSF/serum albumin ratio was highly correlated with MRI measurements of BBB permeability, including the overall mean transfer coefficient and the NAWM BBB permeability hotspot percentage (Pearson's *r* 0.599, *p *= 0.04, and 0.756, *p *= 0.004, respectively). (Figure S6).

## 4 DISCUSSION

In this double blind randomized controlled trial, we found focal hotspots of increased microglial activation and increased BBB permeability in patients with SVD, consistent with previous studies. However, minocycline, which has previously been shown to attenuate both processes in a rodent model of SVD,^13^ had no treatment effect. Our results were consistent across both co‐primary endpoints and a variety of secondary outcomes. Furthermore, we found no effect of minocycline on a panel of blood biomarkers related to endothelial activation and vascular inflammation.

Our study demonstrates the feasibility of using imaging markers of neuroinflammation to assess therapeutic interventions for SVD. The ^11^C‐PK11195 PET signal was highly reproducible between timepoints. In contrast, we found significant variation in BBB permeability measurements between the two time points. DCE‐MRI measurements of BBB permeability correlate with the CSF/serum albumin ratio, the gold standard in vivo BBB measurement,^11^ and we confirmed this in a subset of participants who also had CSF measurements. However, our data suggest that BBB permeability may fluctuate temporally; a previous study using repeated DCE‐MRI found little overlap between areas of increased permeability between scans.^23^ This temporal variation is likely to reduce the power of DCE‐MRI to detect treatment effects.

In addition to the lack of treatment effect on microglial activation, we found no treatment effect on circulating systemic inflammation, either serum hsCRP, or a proteomics panel reflective of endothelial activation and vascular inflammation. This panel is related to disease severity in SVD,^24^ and includes specific MMPs that were reduced in the minocycline group in a rat model.^13^ In particular, serum MMP‐9 levels were not altered with minocycline treatment. MMP‐9 is associated with ECM remodeling and discriminates well between patients with vascular cognitive impairment and Alzheimer's disease,^5^ but it is possible that measurement in the serum is not sensitive enough to capture a treatment effect. Future studies should consider serial measurements in cerebrospinal fluid.

There are[Fig alz13830-fig-0005] a number of reasons why we were not able to replicate the robust treatment effect described in the rodent model. There are no ideal models of SVD, and each only mimics some aspects of the disease.^25^ The model used to demonstrate minocycline efficacy reproduced white matter ischemia by large artery occlusion, and therefore differs significantly from the situation in man where small artery pathology is associated with the white matter changes. Alternative preclinical models of SVD can be investigated using more chronic hypoperfusion, and minocycline was also associated with a reduction in microglia and better white matter integrity in a bilateral carotid occlusion model^26^; however, this also involves intervention to the carotid arteries. Similarly, rodent models of chronic cerebral hypoperfusion can be produced from transgenic animals designed to mimic monogenic forms of SVD but may not involve the same pathophysiological mechanisms.^27^ One of the principal difficulties in translational research is dealing with the heterogeneity in humans relative to animal subjects; the participants in our study had a wide age range and diverse medical histories. It is possible that reducing the heterogeneity of the study sample, for example, by imposing a more restrictive age range, would have increased the likelihood of demonstrating a significant treatment effect (although this may have compromised recruitment).

A further strategy to enrich the study population would have been to select only those participants that showed significant volumes of hotspot tissue either for microglial signal or for BBB permeability. This may also have limited recruitment and rendered the intervention less feasible as a future therapeutic option (i.e., if advanced imaging was required before eligibility could be confirmed); however, ongoing work is assessing whether blood biomarkers and immunophenotyping of peripheral blood may allow us to stratify participants for inclusion in future trials.

In the model, animals were treated with 50 mg/kg minocycline intraperitoneally on alternate days,^13^ which is considerably higher than the clinically licensed dose that we used. Of note, in a 2‐year trial in Alzheimer's disease,^28^ of a group taking 400 mg only 28.8% completed a 2‐year course, suggesting higher doses of minocycline are unlikely to be tolerated. Alternative therapeutic options might replicate some of the effects of minocycline that are proposed to be beneficial in the rodent model; for example, several small molecules and biologic MMP inhibitors are in development,^29^ but none are currently licensed in humans apart from other members of the tetracycline family.

There were small and non‐statistically significant differences between baseline and follow‐up ^11^C‐PK11195 binding (which reduced during the study) and the calculated BBB permeability constant (which increased slightly). It is possible that the participants we recruited were not in a stable phase of SVD; however, given that we aimed to recruit participants at least 3 months post stroke, which should be sufficient for the acute inflammatory response to resolve^30^, ^31^ and that the mean recruitment was nearly 2 years after stroke, it is reasonable to assume our intervention was during the chronic stage of the disease.

Our study has a number of strengths. It was double‐blind, with previously validated endpoints,^6^ and the full sample size and follow‐up period was achieved despite the COVID‐19 pandemic. However, there are also limitations. Although ^11^C‐PK11195 PET is widely used as a marker of microglial activation, it may be confounded by off‐target and non‐specific tissue binding^32^; however, we controlled for these where possible by including endothelial binding in the analysis. A recent transcriptomic study has suggested that TSPO relates to microglial concentration rather than phenotype.^33^ Microglial activation is the endpoint of multiple convergent inflammatory pathways,^34^ and these measurements may not capture more subtle immunomodulatory effects of minocycline in humans, including metalloprotease activity, T cell cytokine production, and neutrophil chemotaxis.^35^ Additionally, while minocycline interacts directly with enzymatic targets within the first 24 h of administration,^36^ it also exerts longer term effects via inhibition of the pro‐inflammatory transcription factor NF‐κB^37^; therefore, it is possible that in humans a beneficial effect could be measured after a treatment period longer than 3 months.

Our sample size was calculated based on observational data using the same neuroimaging protocol; in the interventional phase of our study, the calculated BBB permeability constant had a standard deviation that was twice as large and this may have resulted in the study being underpowered to detect a treatment effect on blood brain barrier permeability. The standard deviation of ^11^C‐PK11195 binding in the interventional cohort was lower than in the observational study.

However, our study was also limited by incomplete imaging data as several participants were unable to have contrast injection due to a decline in renal function between screening and enrolment. Non‐contrast based imaging of the BBB^38^ would help to reduce this limitation and extend recruitment to patients with renal disease that represent a significant proportion of patients with SVD.^39^ In conclusion, our study suggests that minocycline, at clinical doses tolerated in humans and taken for 12 weeks, does not reduce the increased inflammation or BBB permeability seen in SVD, and does not support larger phase III trials of minocycline in sporadic SVD. It is possible that the duration of minocycline treatment was too short to have a measurable therapeutic response. Our results do not exclude a role of inflammation and increased BBB permeability in SVD, but whether these processes are indeed casual or secondary to tissue damage remains to be determined. Further information on this aspect will be provided by the 1‐year follow up in MINERVA which includes MRI DTI imaging and repeat neuropsychometric testing; this will allow determination of whether increased BBB permeability or ^11^C‐PK11195 PET at baseline predicts future tissue damage and cognitive decline, but ultimately causality will only be verified by interventional studies.

## CONFLICT OF INTEREST STATEMENT

The authors report no personal, professional, or financial relationships that would constitute a potential conflict of interest. Author disclosures are available in the supporting information.

## CONSENT STATEMENT

All participants provided written informed consent prior to any trial interventions.

## Supporting information

Supporting Information

Supporting Information

## Data Availability

All study related documents (e.g., study protocol, statistical analysis plan, informed consent form) will be made available to researchers on contact with the corresponding authors from the date of publication. Deidentified participant data will be made available to interested researchers after approval of a proposal, with a signed data access agreement.
