# Supplementary material for: MINocyclinE to Reduce inflammation and blood‐brain barrier leakage in small Vessel diseAse (MINERVA): A phase II, randomized, double‐blind, placebo‐controlled experimental medicine trial
Source: Alzheimers Dement. 2024 Apr 17;20(6):3852–63. doi: 10.1002/alz.13830 (PMC11180856; doi:10.1002/alz.13830)
Supplement: Supplementary file 1 — Supporting Information [file ALZ-20-3852-s002.docx]

**supplemMINocyclinE to Reduce inflammation and blood-brain barrier leakage in small Vessel diseAse (MINERVA): a phase II, randomised, double-blind, placebo-controlled experimental medicine trial**

**Supplementary Material**

Robin B Brown^1^, Daniel J Tozer^1^, Laurence Loubière^1^, Eric L Harshfield^1^, Young T Hong^1,2^, Tim D Fryer^1,2^, Guy B Williams^1,2^, Martin J Graves^3^, Franklin I Aigbirhio^1,2^, John T O’Brien^4^ & Hugh S Markus^1^

^1^Department of Clinical Neurosciences, University of Cambridge, Hills Road, Cambridge, UK. CB2 0QQ

^2^Wolfson Brain Imaging Centre, University of Cambridge, Hills Road, Cambridge, UK. CB2 0QQ

^3^Department of Radiology, University of Cambridge, Hills Road, Cambridge, Cambridge, UK. CB2 0QQ

^4^Department of Psychiatry, University of Cambridge, Hills Road, Cambridge, UK. CB2 0QQ

Supplementary Figure 1. Inclusion and exclusion criteria

Supplementary Table 1. MRI sequence details

Supplementary Methods/Results 1. Validation of DCE-MRI

Supplementary Figure 2: Mean ^11^C-PK11195 binding potential (BPND) in normal appearing white matter at baseline and follow-up.

Supplementary Figure 3: Mean BBB transfer constant (Ki) in normal appearing white matter at baseline and follow-up.

Supplementary Table 2: Difference in baseline and follow-up measurements of ^11^C-PK11195 binding

Supplementary Table 3: Difference in baseline and follow-up measurements of K_i_

Supplementary Table 4: Effect of treatment on serum CRP

Supplementary Figure 4: Screeplot of eigenvalues from PCA of proteomic panel

Supplementary Table 5: Effect of treatment on first three principal components of proteomics panel

Supplementary Table 6: Effect of treatment on individual biomarkers in proteomics panel

Supplementary Table 7: Effect of treatment on BBB/PET outcomes based on non-eroded white matter masks

Supplementary Figure 5: Graphical representation of per-protocol cohort stratified by age, sex and disease severity (white matter hyperintensity (WMH) volume)

Supplementary Table 8: Participant characteristics (per-protocol cohort)

Supplementary Table 9: Primary outcome results (per-protocol cohort)

Supplementary Table 10: Treatment effect on CRP (per-protocol cohort)

Supplementary Table 11: Treatment effect on first three principal components of proteomics panel (per-protocol cohort)

Supplementary Table 12: Adverse effects, intention-to-treat population

Supplementary Figure 6. DCE-MRI outcomes vs CSF/serum albumin ratio

**
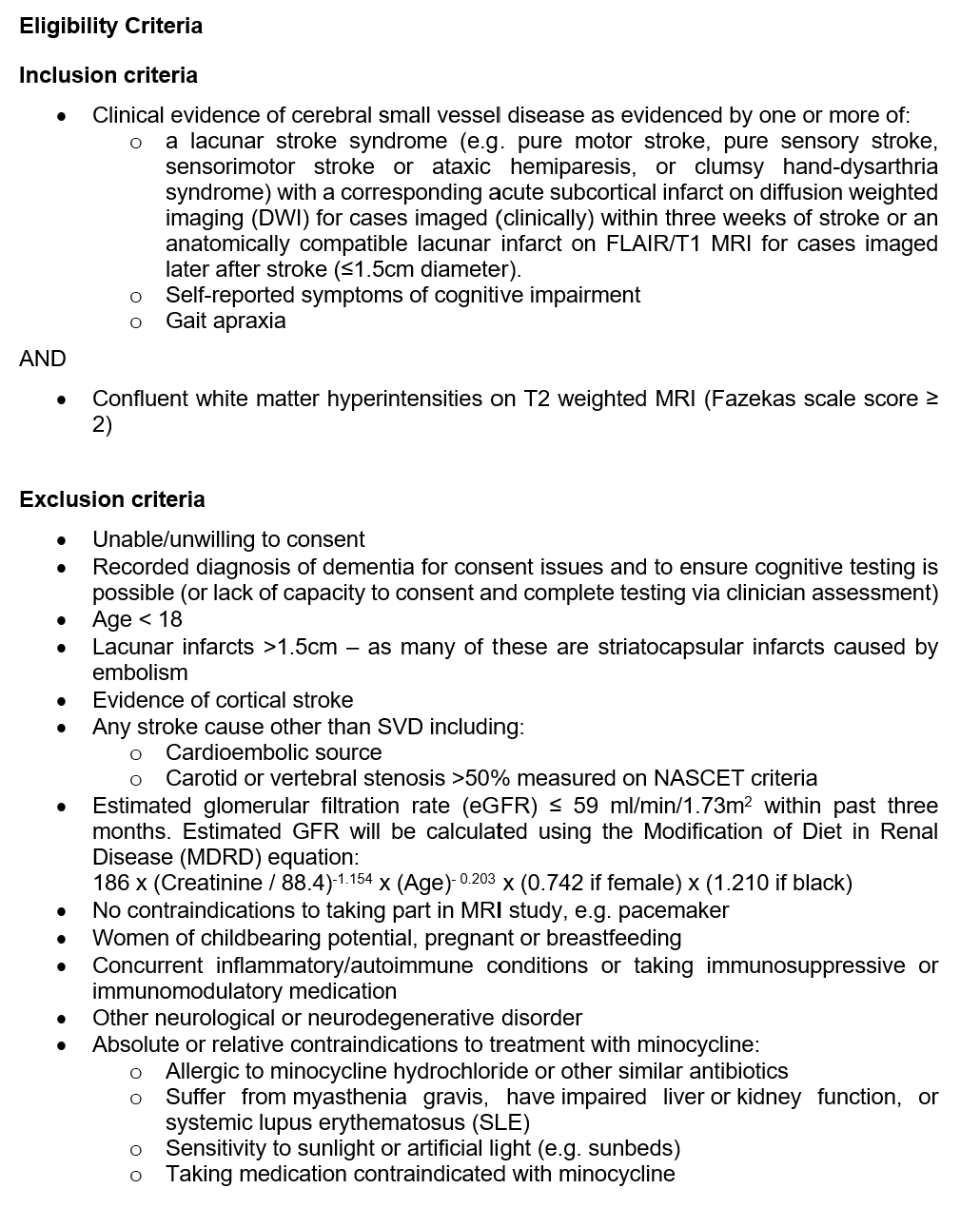
Figure S.1. MINERVA trial inclusion and exclusion criteria (reproduced from Brown RB, Tozer DJ, Loubiere LH et al., *Eur Stroke J* 2022 under a Creative Commons Attribution 4.0 License).**

**Table S.1. MRI Sequence details**

| **Sequence** | **Acquisition parameters** |
| --- | --- |
| T_1_-weighted | Axial 3D fast-spoiled gradient echo sequence (BRAVO), flip angle = 12°, inversion time = 450 ms, field of view =28 mm, slice thickness = 1 mm, number of slices = 192, reconstructed matrix size = 512 × 512 |
| T_2_-weighted | Axial T_2_ fast spin echo sequence angled anterior commissure-posterior commissure (AC-PC), flip angle = 111°, TE = 85 ms, TR = 6000 ms, field of view = 22 mm, slice thickness = 5 mm, number of slices = 31, reconstructed matrix size = 1024 × 1024 |
| FLAIR | Axial T_2_ FLAIR, angled AC-PC, flip angle = 160°, TR = 8800 ms, TE = 120 ms, TI = 2445 ms, field of view = 22 cm, slice thickness = 5 mm, number of slices = 28, reconstructed matrix size = 256 × 256 |
| Susceptibility-weighted | Axial susceptibility weighted imaging, flip angle = 17°, repetition time = 40.6 ms, echo time = 24.2 ms, field of view = 22 cm, slice thickness = 2 mm, number of slices = 70, reconstructed matrix size = 256 × 256 |
| DTI | Axial DTI, angled anterior commissure-posterior commissure with the diffusion gradients applied in 63 directions with a b-value = 1000 s/mm^2^, TE = minimum, TR = 15763ms, field of view = 19.2 cm, slice thickness = 2 mm, number of slices = 65-70 depending on slice angulation reconstructed matrix size = 256 × 256 |
| DCE-MRI | 3D radiofrequency (RF) spoiled gradient echo, TR = 6.3 ms, TE = 1.8 ms, number of slices = 16, reconstructed matrix size = 256 × 256, final resolution = 0.94 × 0.94 × 3mm, flip angles = 2°,5°,12°,17°,22°, and 27°, temporal resolution = 15 seconds per flip angle with interphase interval of 15 seconds (eight cycles) |

**Validation of DCE-MRI using CSF/serum albumin ratio**

The following details supplementary methods and results for the CSF sub-study.

**Methods**

All patients enrolled in the trial were asked if they would additionally give a sample of cerebrospinal fluid (CSF), taken via a lumbar puncture (LP). If they consented, LP was performed at the baseline appointment (after the MRI scan, to avoid any spurious increases in BBB permeability introduced by the procedure).

10ml CSF was centrifuged at 2000g for 10 minutes. The supernatant was aliquoted into 2ml Eppendorf tubes and stored at -80°C for *en bloc* analysis. CSF albumin was measured at the University of Cambridge Core Biochemical Assay Laboratory by ELISA using a Siemens Dimension EXL auto-analyser. To allow calculation of the CSF/serum albumin ratio, CSF samples were sent with a paired aliquot of serum which was also quantified using ELISA by the same method. The CSF/serum ratio was calculated in mg/g.

**Reproducibility of imaging data**

^11^C-PK11195 BP_ND_ in NAWM was highly correlated between timepoints (Pearson’s r 0.785, 95% CI 0.608-0.887; see Figure S.2). There were small and non-significant differences between the BP_ND_ at baseline and follow-up in the placebo group. Table S.2 shows the differences between baseline and follow-up imaging values for the primary outcome (hotspots as percentage of NAWM) and mean BP_ND_ in eroded NAWM.

**Table S.2. Difference in baseline and follow-up measurements of ^11^C-PK11195 binding**

|  | **Baseline** | **Follow-up** | **p-value** (student’s paired t-test) |
| --- | --- | --- | --- |
| **Hotspot (%NAWM)** |  |  |  |
| Minocycline group | 10.71 ± 4.04 | 9.97 ± 5.50 | 0.45 |
| Placebo group | 10.11 ± 4.67 | 7.79 ± 5.67 | 0.19 |
| Overall trial cohort | 10.45 ± 4.27 | 8.94 ± 5.60 | 0.14 |
| **Mean BP_ND_** |  |  |  |
| Minocycline group | -0.036 ± 0.025 | -0.032 ± 0.032 | 0.97 |
| Placebo group | -0.041 ± 0.024 | -0.028 ± 0.027 | 0.13 |
| Overall trial cohort | -0.038 ± 0.024 | -0.030 ± 0.029 | 0.31 |

Mean BBB transfer constant (K_i_) values in the NAWM were poorly correlated between baseline and follow-up scans (Pearson’s r -0.089, 95% CI -0.430-0.274; see Figure S.3). There were small and non-significant differences between the BBB permeability constant at baseline and follow-up in the placebo group. Table S.3 shows the differences between baseline and follow-up imaging values for the primary outcome (hotspots as percentage of NAWM) and mean K_i_ in eroded NAWM.

**Table S.3. Difference in baseline and follow-up measurements of K_i_**

|  | **Baseline** | **Follow-up** | **p-value** (student’s paired t-test) |
| --- | --- | --- | --- |
| **Hotspot (%NAWM)** |  |  |  |
| Minocycline group | 4.08 ± 3.69 | 6.19 ± 5.09 | 0.26 |
| Placebo group | 8.49 ± 8.45 | 13.04 ± 9.24 | 0.08 |
| Overall trial cohort | 6.22 ± 6.73 | 9.41 ± 8.01 | 0.12 |
| **Mean K_i_ (ml/g/min)** |  |  |  |
| Minocycline group | 0.0002 ± 0.0003 | 0.0002 ± 0.0003 | 0.96 |
| Placebo group | 0.0008 ± 0.0001 | 0.0005 ± 0.0004 | 0.69 |
| Overall trial cohort | 0.0005 ± 0.0002 | 0.0004 ± 0.0003 | 0.68 |

**Figure S.2. Mean ^11^C-PK11195 binding potential (BP_ND_) in normal appearing white matter at baseline and follow-up.**

**
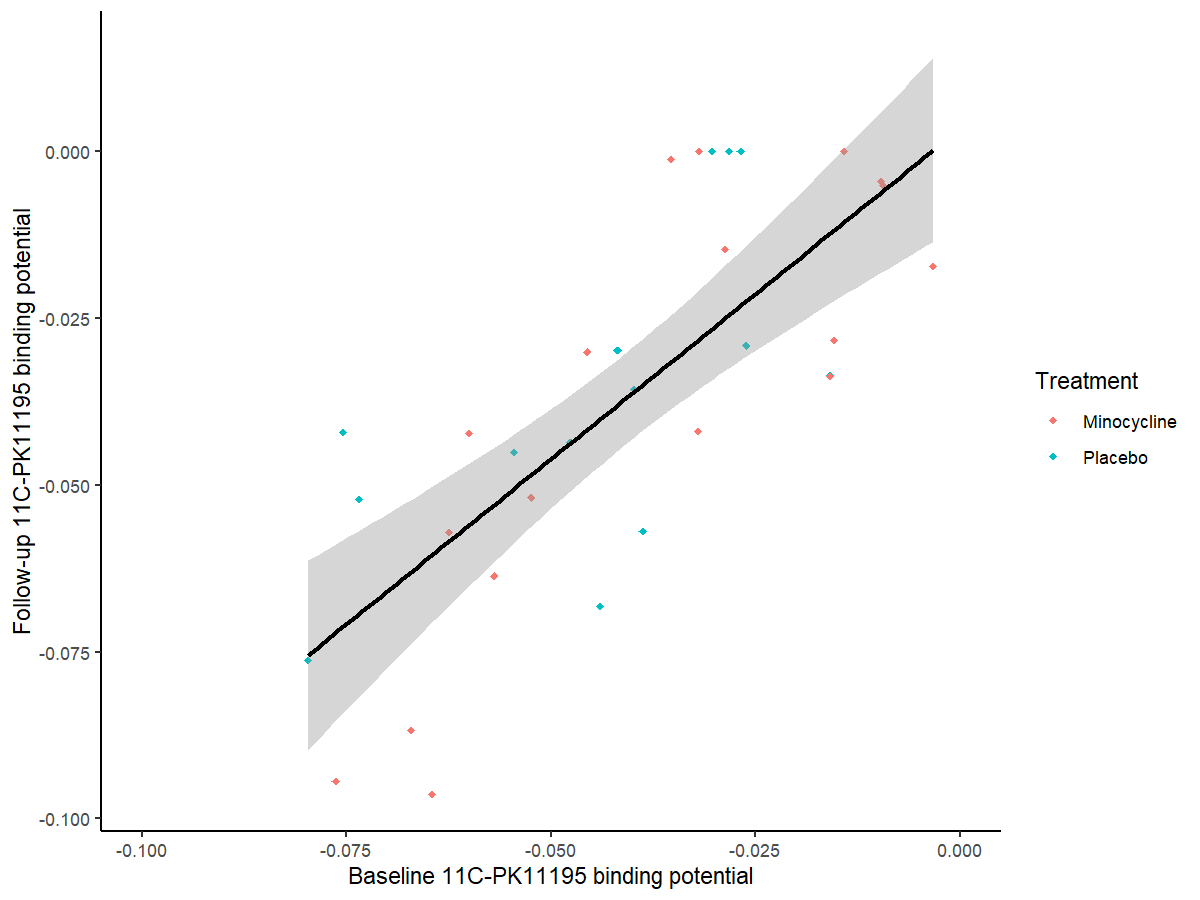
.**

**
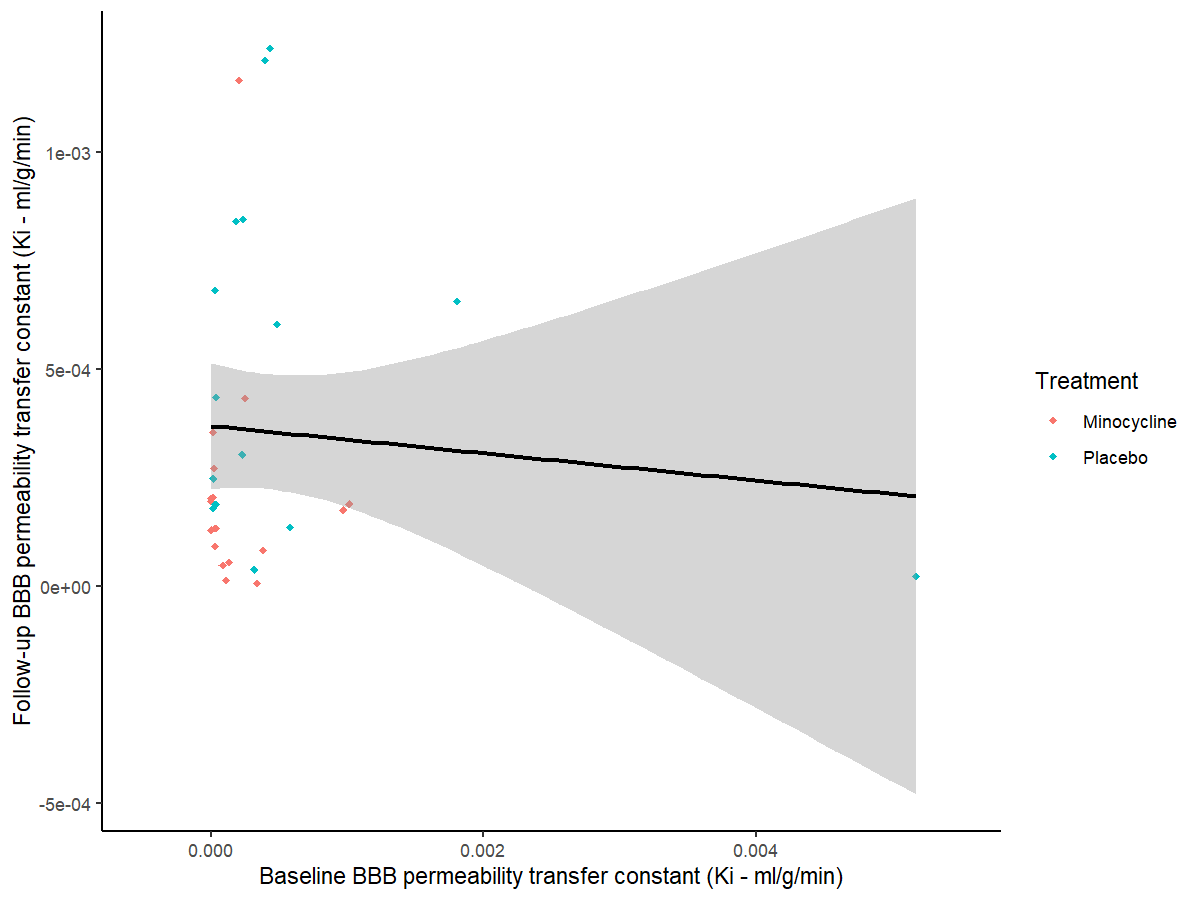
Figure S.3. Mean BBB transfer constant (K_i_) in normal appearing white matter at baseline and follow-up.**

**Effect of treatment on serum biomarkers**

**Table S.4. Effect of treatment on CRP in intention-to-treat population.**

|  | **RR (95% CI)** | ***P*-value** |
| --- | --- | --- |
| **Secondary analyses in intention-to-treat population** |  |  |
| Change in CRP | 30.67 (0.01-65890.4) | 0.37 |
| Adjusted for age | 42.83 (0.01-146654.2) | 0.36 |

**Figure S.4: Screeplot of eigenvalues from principal component analysis of protein measurements.**


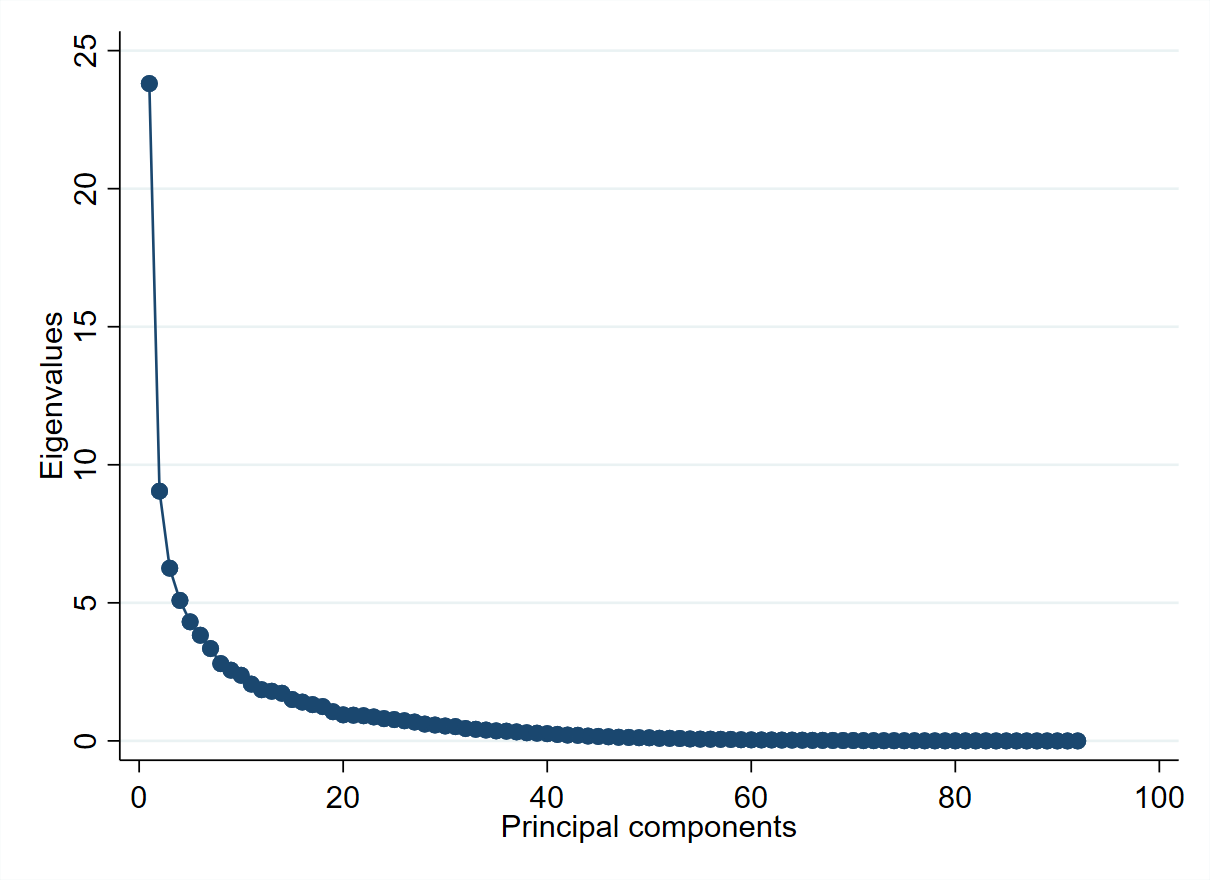


**Table S.5. Effect of treatment on first three principal components of protein measurements in intention-to-treat population.**

|  | **RR (95% CI)** | ***P*-value** |
| --- | --- | --- |
| **Secondary analyses in intention-to-treat population** |  |  |
| Change in first principal component of protein levels | 5.57 (0.45-68.92) | 0.18 |
| Adjusted for age | 5.80 (0.40-83.87) | 0.19 |
|  |  |  |
| Change in second principal component of protein levels | 2.00 (0.25-15.71) | 0.50 |
| Adjusted for age | 2.18 (0.24-19.42) | 0.48 |
|  |  |  |
| Change in third principal component of protein levels | 2.67 (0.40-17.97) | 0.30 |
| Adjusted for age | 2.71 (0.36-20.50) | 0.33 |

**Table S.6. Effect of treatment on protein levels in modified intention-to-treat population.**

|  | **Unadjusted** | | **Adjusted for age** | |
| --- | --- | --- | --- | --- |
| **Protein name** | **RR (95% CI)** | ***P*-value** | **RR (95% CI)** | ***P*-value** |
| TNFRSF14 | 1.09 (0.84-1.40) | 0.51 | 1.08 (0.82-1.41) | 0.57 |
| LDLreceptor | 1.35 (1.00-1.82) | 0.05 | 1.41 (1.03-1.93) | 0.03 |
| ITGB2 | 1.22 (0.88-1.68) | 0.22 | 1.26 (0.90-1.77) | 0.18 |
| IL17RA | 0.97 (0.78-1.21) | 0.80 | 0.97 (0.77-1.23) | 0.81 |
| TNFR2 | 1.25 (0.99-1.59) | 0.06 | 1.26 (0.98-1.62) | 0.07 |
| MMP9 | 1.60 (0.97-2.65) | 0.07 | 1.64 (0.96-2.80) | 0.07 |
| EPHB4 | 1.06 (0.89-1.26) | 0.48 | 1.05 (0.88-1.27) | 0.57 |
| IL2RA | 1.41 (1.09-1.83) | 0.01 | 1.43 (1.09-1.88) | 0.01 |
| OPG | 1.03 (0.88-1.21) | 0.72 | 1.03 (0.87-1.22) | 0.72 |
| ALCAM | 1.11 (0.94-1.31) | 0.21 | 1.11 (0.93-1.32) | 0.23 |
| TFF3 | 0.97 (0.82-1.14) | 0.71 | 0.96 (0.81-1.14) | 0.64 |
| SELP | 1.00 (0.66-1.50) | 0.99 | 1.04 (0.67-1.60) | 0.87 |
| CSTB | 0.91 (0.57-1.45) | 0.68 | 0.85 (0.52-1.39) | 0.51 |
| MCP1 | 1.05 (0.72-1.52) | 0.81 | 1.14 (0.78-1.67) | 0.49 |
| CD163 | 1.32 (1.06-1.66) | 0.02 | 1.35 (1.06-1.71) | 0.02 |
| Gal3 | 1.19 (0.91-1.55) | 0.19 | 1.14 (0.87-1.51) | 0.33 |
| GRN | 1.08 (0.90-1.30) | 0.38 | 1.09 (0.90-1.32) | 0.39 |
| NTproBNP | 1.06 (0.78-1.44) | 0.71 | 1.02 (0.74-1.42) | 0.88 |
| BLM hydrolase | 1.08 (0.76-1.53) | 0.67 | 1.00 (0.70-1.44) | 0.98 |
| PLC | 1.02 (0.88-1.18) | 0.84 | 1.03 (0.88-1.21) | 0.67 |
| LTBR | 1.15 (0.97-1.37) | 0.10 | 1.14 (0.95-1.37) | 0.14 |
| Notch3 | 1.07 (0.87-1.31) | 0.53 | 1.09 (0.88-1.35) | 0.43 |
| TIMP4 | 0.97 (0.76-1.23) | 0.78 | 1.00 (0.78-1.29) | 1.00 |
| CNTN1 | 0.95 (0.79-1.14) | 0.56 | 0.97 (0.8-1.17) | 0.72 |
| CDH5 | 1.07 (0.89-1.27) | 0.47 | 1.06 (0.88-1.28) | 0.53 |
| TLT2 | 0.96 (0.74-1.23) | 0.73 | 0.94 (0.72-1.23) | 0.67 |
| FABP4 | 1.43 (1.01-2.02) | 0.04 | 1.45 (1.01-2.10) | 0.04 |
| TFPI | 1.13 (0.87-1.48) | 0.34 | 1.20 (0.91-1.58) | 0.20 |
| PAI | 1.06 (0.88-1.27) | 0.57 | 1.09 (0.89-1.32) | 0.39 |
| CCL24 | 1.37 (1.06-1.76) | 0.02 | 1.39 (1.06-1.83) | 0.02 |
| TR | 1.13 (0.93-1.38) | 0.22 | 1.13 (0.92-1.40) | 0.23 |
| TNFRSF10C | 1.11 (0.91-1.37) | 0.30 | 1.11 (0.90-1.39) | 0.32 |
| GDF15 | 1.16 (0.89-1.50) | 0.27 | 1.14 (0.87-1.51) | 0.33 |
| SELE | 0.95 (0.79-1.15) | 0.61 | 0.97 (0.80-1.18) | 0.74 |
| AZU1 | 1.63 (0.68-3.91) | 0.26 | 1.68 (0.67-4.25) | 0.26 |
| DLK1 | 1.14 (0.96-1.35) | 0.14 | 1.15 (0.95-1.38) | 0.14 |
| SPON1 | 1.12 (0.96-1.30) | 0.15 | 1.10 (0.94-1.29) | 0.23 |
| MPO | 1.41 (0.84-2.36) | 0.19 | 1.44 (0.83-2.48) | 0.19 |
| CXCL16 | 1.15 (0.99-1.34) | 0.06 | 1.17 (1.00-1.37) | 0.05 |
| IL6RA | 1.02 (0.88-1.17) | 0.81 | 1.02 (0.88-1.19) | 0.78 |
| RETN | 1.05 (0.71-1.57) | 0.79 | 1.08 (0.71-1.65) | 0.71 |
| IGFBP1 | 1.88 (1.01-3.53) | 0.05 | 1.68 (0.87-3.23) | 0.12 |
| CHIT1 | 1.24 (0.90-1.72) | 0.19 | 1.25 (0.89-1.77) | 0.19 |
| TRAP | 1.02 (0.85-1.23) | 0.81 | 1.03 (0.84-1.25) | 0.79 |
| GP6 | 0.91 (0.54-1.52) | 0.71 | 0.86 (0.50-1.49) | 0.59 |
| PSPD | 1.36 (1.02-1.83) | 0.04 | 1.36 (1.00-1.87) | 0.05 |
| PI3 | 0.85 (0.65-1.10) | 0.20 | 0.86 (0.65-1.13) | 0.27 |
| EpCAM | 1.34 (0.85-2.12) | 0.20 | 1.33 (0.82-2.16) | 0.24 |
| APN | 1.10 (0.96-1.26) | 0.17 | 1.10 (0.96-1.27) | 0.17 |
| AXL | 1.16 (0.96-1.40) | 0.12 | 1.17 (0.96-1.42) | 0.12 |
| IL1RT1 | 1.09 (0.93-1.29) | 0.29 | 1.08 (0.91-1.29) | 0.37 |
| MMP2 | 1.18 (1.01-1.38) | 0.04 | 1.18 (1.00-1.40) | 0.05 |
| FAS | 1.28 (1.06-1.54) | 0.01 | 1.25 (1.03-1.53) | 0.03 |
| MB | 1.42 (1.03-1.96) | 0.03 | 1.39 (0.99-1.95) | 0.06 |
| TNFSF13B | 1.03 (0.88-1.22) | 0.68 | 1.05 (0.88-1.26) | 0.55 |
| PRTN3 | 1.23 (0.63-2.38) | 0.54 | 1.27 (0.63-2.55) | 0.50 |
| PCSK9 | 1.02 (0.80-1.29) | 0.88 | 1.06 (0.83-1.36) | 0.62 |
| UPAR | 1.15 (0.80-1.66) | 0.43 | 1.19 (0.81-1.74) | 0.38 |
| OPN | 1.22 (0.94-1.59) | 0.13 | 1.19 (0.90-1.57) | 0.21 |
| CTSD | 1.01 (0.83-1.22) | 0.91 | 1.05 (0.86-1.28) | 0.64 |
| PGLYRP1 | 1.06 (0.67-1.66) | 0.80 | 1.09 (0.68-1.75) | 0.72 |
| CPA1 | 1.47 (1.06-2.05) | 0.02 | 1.36 (0.97-1.91) | 0.07 |
| JAMA | 0.97 (0.75-1.25) | 0.79 | 0.94 (0.71-1.23) | 0.62 |
| Gal4 | 0.96 (0.77-1.21) | 0.74 | 0.96 (0.75-1.22) | 0.74 |
| IL1RT2 | 1.03 (0.89-1.20) | 0.67 | 1.04 (0.89-1.22) | 0.58 |
| SHPS1 | 1.15 (1.00-1.31) | 0.04 | 1.16 (1.00-1.33) | 0.05 |
| CCL15 | 1.20 (0.98-1.46) | 0.08 | 1.22 (0.98-1.50) | 0.07 |
| CASP3 | 1.77 (0.87-3.60) | 0.11 | 1.48 (0.71-3.07) | 0.28 |
| uPA | 1.08 (0.85-1.36) | 0.54 | 1.11 (0.86-1.42) | 0.42 |
| CPB1 | 1.35 (1.03-1.77) | 0.03 | 1.33 (1.00-1.78) | 0.05 |
| CHI3L1 | 1.28 (0.85-1.95) | 0.23 | 1.44 (0.95-2.21) | 0.09 |
| ST2 | 1.26 (0.98-1.62) | 0.07 | 1.28 (0.98-1.67) | 0.07 |
| tPA | 1.24 (0.97-1.58) | 0.09 | 1.22 (0.94-1.58) | 0.14 |
| SCGB3A2 | 1.31 (1.07-1.61) | 0.01 | 1.27 (1.03-1.58) | 0.03 |
| EGFR | 1.05 (0.90-1.23) | 0.50 | 1.06 (0.90-1.25) | 0.47 |
| IGFBP7 | 1.12 (0.91-1.39) | 0.28 | 1.12 (0.89-1.41) | 0.32 |
| CD93 | 1.02 (0.87-1.18) | 0.83 | 1.04 (0.88-1.22) | 0.64 |
| IL18BP | 1.09 (0.92-1.30) | 0.31 | 1.09 (0.91-1.31) | 0.33 |
| COL1A1 | 1.00 (0.81-1.24) | 0.99 | 1.04 (0.84-1.30) | 0.69 |
| PON3 | 0.96 (0.71-1.30) | 0.80 | 0.97 (0.71-1.34) | 0.86 |
| CTSZ | 1.04 (0.86-1.25) | 0.70 | 1.06 (0.87-1.30) | 0.54 |
| MMP3 | 0.97 (0.73-1.29) | 0.84 | 0.99 (0.73-1.33) | 0.93 |
| RARRES2 | 1.07 (0.94-1.22) | 0.28 | 1.06 (0.92-1.22) | 0.41 |
| ICAM2 | 0.99 (0.84-1.17) | 0.95 | 1.00 (0.84-1.19) | 0.96 |
| KLK6 | 0.95 (0.74-1.21) | 0.65 | 0.95 (0.73-1.23) | 0.69 |
| PDGFsubunitA | 1.17 (0.91-1.50) | 0.21 | 1.17 (0.90-1.53) | 0.23 |
| TNFR1 | 1.16 (0.97-1.39) | 0.10 | 1.18 (0.98-1.43) | 0.08 |
| IGFBP2 | 1.16 (0.89-1.51) | 0.26 | 1.14 (0.86-1.50) | 0.36 |
| vWF | 1.26 (0.96-1.66) | 0.10 | 1.22 (0.91-1.62) | 0.18 |
| PECAM1 | 1.03 (0.86-1.23) | 0.77 | 1.00 (0.83-1.22) | 0.96 |
| MEPE | 1.00 (0.83-1.21) | 0.99 | 1.01 (0.83-1.23) | 0.94 |
| CCL16 | 1.23 (0.97-1.57) | 0.09 | 1.21 (0.94-1.57) | 0.13 |

**Outcomes based on non-eroded white matter**

**Table S.7. Effect of minocycline on change in BBB permeability and ^11^C-PK11195 binding hotspot volumes and mean BBB transfer constant (K_i_) and ^11^C-PK11195 binding potential (BP_ND_) values in non-eroded white matter (WM).**

|  | **RR (95% CI)** | ***P*-value** |
| --- | --- | --- |
| **Primary analyses** |  |  |
| Change in BBB permeability hotspot volume in non-eroded WM | 0.96 (0.90-1.02) | 0.22 |
| Adjusted for age | 0.97 (0.90-1.03) | 0.31 |
| Change in ^11^C-PK11195 binding hotspot volume in non-eroded WM | 1.01 (0.98-1.03) | 0.55 |
| Adjusted for age | 1.01 (0.98-1.04) | 0.59 |
| **Secondary analyses** |  |  |
| Change in mean K_i_ in non-eroded WM | 1.00 (1.00-1.00) | 0.61 |
| Adjusted for age | 1.00 (1.00-1.00) | 0.76 |
| Change in mean ^11^C-PK11195 BP_ND_ in non-eroded WM | 0.99 (0.98-1.01) | 0.43 |
| Adjusted for age | 0.99 (0.97-1.01) | 0.40 |
|  |  |  |
|  |  |  |
|  |  |  |
|  |  |  |
|  |  |  |
|  |  |  |
|  |  |  |
|  |  |  |
|  |  |  |
|  |  |  |

**Results based on per-protocol analysis**

Four participants did not complete treatment due to side effects (two) or unrelated health conditions (two), leaving 40 participants included in the per-protocol analysis. Figure S.5 shows the updated cohort graphic including only these individuals.

**
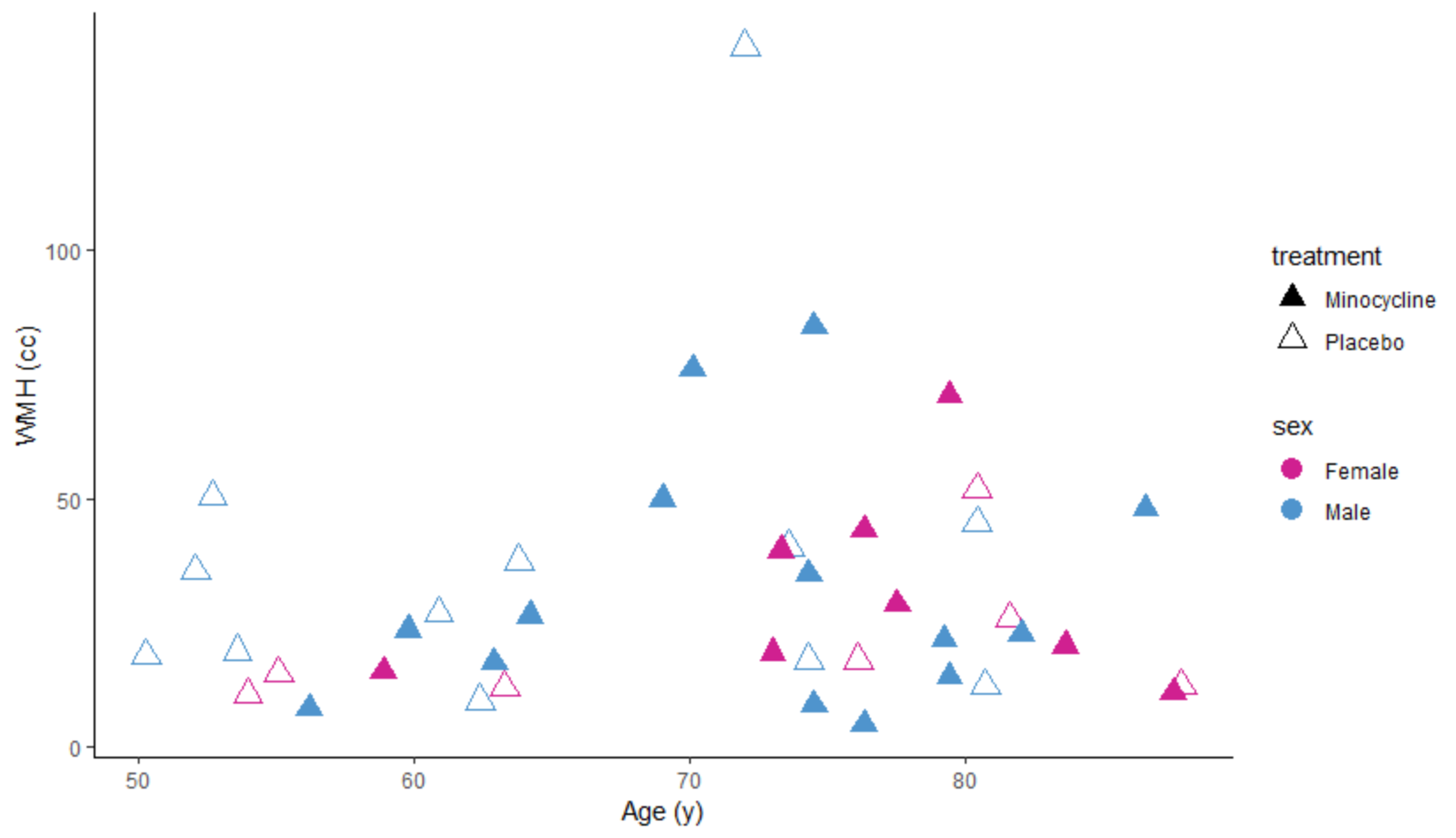
Figure S.5.** **Graphical representation of per-protocol cohort stratified by age, sex and disease severity (white matter hyperintensity (WMH) volume)**

Table S.8 shows the between group comparison in the per-protocol population. Note that in the per-protocol population the difference in mean age between the groups was not statistically significant and subsequent analyses are therefore presented unadjusted with sensitivity analyses based on sub-populations above and below median age.

**Table S.8. Between group comparisons in the per-protocol analysis. Values are mean (SD) or median [IQR]. P-value is from t-test, χ-square test (or Fisher’s exact test if frequency is less than 5 for any comparison), or quantile regression of medians, as appropriate.**

|  | **Minocycline** | **Placebo** | **Overall** | ***p* value** |
| --- | --- | --- | --- | --- |
|  | **(N=19)** | **(N=21)** | **(N=40)** |  |
| Age (years) | 71.8 (9.0) | 66.5 (11.9) | 69.0 (10.8) | 0.12 |
| Sex |  |  |  |  |
| Female | 8 (42.1%) | 7 (33.3%) | 15 (37.5%) | 0.57 |
| Male | 11 (57.9%) | 14 (66.7%) | 25 (62.5%) | |
| Ethnicity - White British | | |  |  |
| Yes | 16 (84.2%) | 19 (90.5%) | 35 (87.5%) | 0.65 |
| No | 3 (15.8%) | 2 (9.5%) | 5 (12.5%) |  |
| Education (years) | 12 [12 – 14] | 14 [12 – 17] | 13 [12 – 17] | 0.17 |
| Hypertension | |  |  |  |
| Yes | 18 (94.7%) | 16 (76.2%) | 34 (85.0%) | 0.19 |
| No | 1 (5.3%) | 5 (23.8%) | 6 (15.0%) |  |
| Hyperlipidaemia | |  |  |  |
| Yes | 12 (63.2%) | 18 (85.7%) | 30 (75.0%) | 0.15 |
| No | 7 (36.8%) | 3 (14.3%) | 10 (25.0%) | |
| Ischaemic heart disease | | |  |  |
| Yes | 2 (10.5%) | 1 (4.8%) | 3 (7.5%) | 0.60 |
| No | 17 (89.5%) | 20 (95.2%) | 37 (92.5%) | |
| Diabetes mellitus | |  |  |  |
| Yes | 1 (5.3%) | 5 (23.8%) | 6 (15.0%) | 0.19 |
| No | 18 (92.7%) | 16 (76.2%) | 34 (85.0%) | |
| Body mass index (kg/m2) | 31.5 (10.7) | 30.2 (7.5) | 30.8 (9.0) | 0.66 |
| Current smoker | |  |  |  |
| Yes | 4 (21.1%) | 2 (9.5%) | 6 (15.0%) | 0.40 |
| No | 15 (79.0%) | 19 (90.5%) | 34 (85.0%) |  |
| Time since stroke (months) | 20.7 (24.1) | 21.1 (20.4) | 20.9 (22.0) | 0.96 |
| WMH(cc) | 30.1 (20.5) | 31.5 (30.0) | 30.8 (25.5) | 0.88 |
| Lacunes | 2 [2 – 3] | 2 [1 – 4] | 2 [1 – 3] | 1.00 |
| CMBs | 0.5 [0 – 3] | 0 [0 – 2] | 0 [0 – 2] | 0.43 |
| Brain volume (cc) | 1433 (85.8) | 1427 (69.2) | 1429 (76.7) | 0.82 |

**Table S.9. Effect of treatment on change in volume and mean transfer constant of hotspots of white matter BBB permeability, and on volume and mean 11C-PK11195 binding of hotspots of microglial activation in normal white matter, in per-protocol population.**

|  | RR (95% CI) | *P*-value | Interaction *P*-value |
| --- | --- | --- | --- |
| **Primary analyses in per-protocol population** |  |  |  |
| Change in BBB permeability hotspot volume | 0.97 (0.91-1.03) | 0.35 |  |
| Change in 11C-PK11195 binding hotspot volume | 1.01 (0.98-1.05) | 0.50 |  |
|  | | | |
| **Subgroup analyses by age in per-protocol population** | | | |
| Change in BBB hotspot volume | 0.97 (0.91-1.03) | 0.35 | 0.43 |
| Age below median | 1.00 (0.88-1.13) | 0.99 |  |
| Age above median | 0.95 (0.90-1.00) | 0.04 |  |
|  | | | |
| Change in 11C-PK11195 binding hotspot volume | 1.01 (0.98-1.05) | 0.50 | **0.02** |
| Age below median | 0.98 (0.93-1.02) | 0.26 |  |
| Age above median | 1.05 (1.00-1.11) | 0.05 |  |

**Table S.10. Effect of treatment on CRP in per-protocol population.**

|  | **RR (95% CI)** | ***P*-value** |
| --- | --- | --- |
| **Secondary analyses in per-protocol population** |  |  |
| Change in CRP | 39.98 (0.01-156150.7) | 0.37 |
| Adjusted for age | 53.36 (0.01-304191.0) | 0.36 |

**Table S.11. Effect of treatment on first three principal components of protein measurements in per-protocol population**

|  | **RR (95% CI)** | ***P*-value** |
| --- | --- | --- |
| **Secondary analyses in per-protocol population** |  |  |
| Change in first principal component of protein levels | 9.30 (0.82-105.7) | 0.07 |
| Adjusted for age | 7.85 (0.62-99.0) | 0.11 |
|  |  |  |
| Change in second principal component of protein levels | 1.94 (0.22-16.82) | 0.54 |
| Adjusted for age | 1.99 (0.21-19.08) | 0.54 |
|  |  |  |
| Change in third principal component of protein levels | 3.79 (0.58-24.93) | 0.16 |
| Adjusted for age | 3.32 (0.47-23.66) | 0.22 |

**Adverse events**

Figure S.12 shows the adverse effect profile stratified by treatment group.

**Table S.12. Adverse effects and serious adverse effects stratified by treatment allocation (intention-to-treat population).**

|  | **Minocycline (*n* = 23)** | **Placebo**  **(*n* = 21)** |
| --- | --- | --- |
| **All adverse events** |  |  |
| Total number of adverse events | 18 | 10 |
| Mean adverse events per patient | 0.782 ± 0.83 | 0.47 ± 0.83 |
| Number of participants with ≥ 1 adverse event | 11 (47.8%) | 7 (33.3%) |
| Number of patients who withdrew from trial due to adverse events | 4 (17.4%) | 0 |
| **Serious adverse events** |  |  |
| Hospitalisation | 2 (8.7%) | 0 |
| Recurrent stroke | 1 (4.3%) | 0 |
| **Expected side effects** |  |  |
| Dizziness | 2 (8.7%) | 4 (19.0%) |
| Nausea | 3 (13.0%) | 0 |
| Vomiting | 1 (4.3%) | 0 |
| Gastro-oesophageal reflux | 6 (26.1%) | 1 (4.8%) |
| Diarrhoea | 2 (8.7%) | 3 (14.2%) |
| Sunburn | 0 | 0 |
| Photosensitivity | 0 | 0 |
| Skin discoloration | 0 | 1 (4.8%) |
| Other potential side effects | 2 (8.7%) | 1 (4.8%) |

**Validation of DCE-MRI**

**Results**

Paired CSF and serum samples were obtained for 12 participants. The mean CSF/serum albumin ratio was 5.39 ± 1.71 mg/g. The CSF/serum albumin ratio was highly correlated with radiological measurements of BBB permeability, including both the overall mean p transfer constant K_i_ and the percentage BBB permeability hotspot volume in the NAWM (Pearson’s *r* correlation coefficients 0.599, *p* = 0.04, and 0.756, *p* = 0.004 respectively). To test the sensitivity of these results, we assessed the relationship between the CSF/serum albumin ratio and the overall mean ant K_i_ and the percentage BBB permeability hotspot volume in the entirety of the white matter; these were also significantly correlated (correlation coefficients 0.626, *p* = 0.03, and 0.709, *p* = 0.010 respectively). These data are shown in Figure S.6.

**Figure S.6. Scatter plots showing correlation of CSF/serum albumin to (A) mean BBB transfer constant (K_i_) in normal appearing white matter (NAWM); (B) percentage BBB permeability hotspot volume in NAWM; (C) mean K_i_ in white matter (WM); (D) percentage BBB permeability hotspot volume in WM.**

**
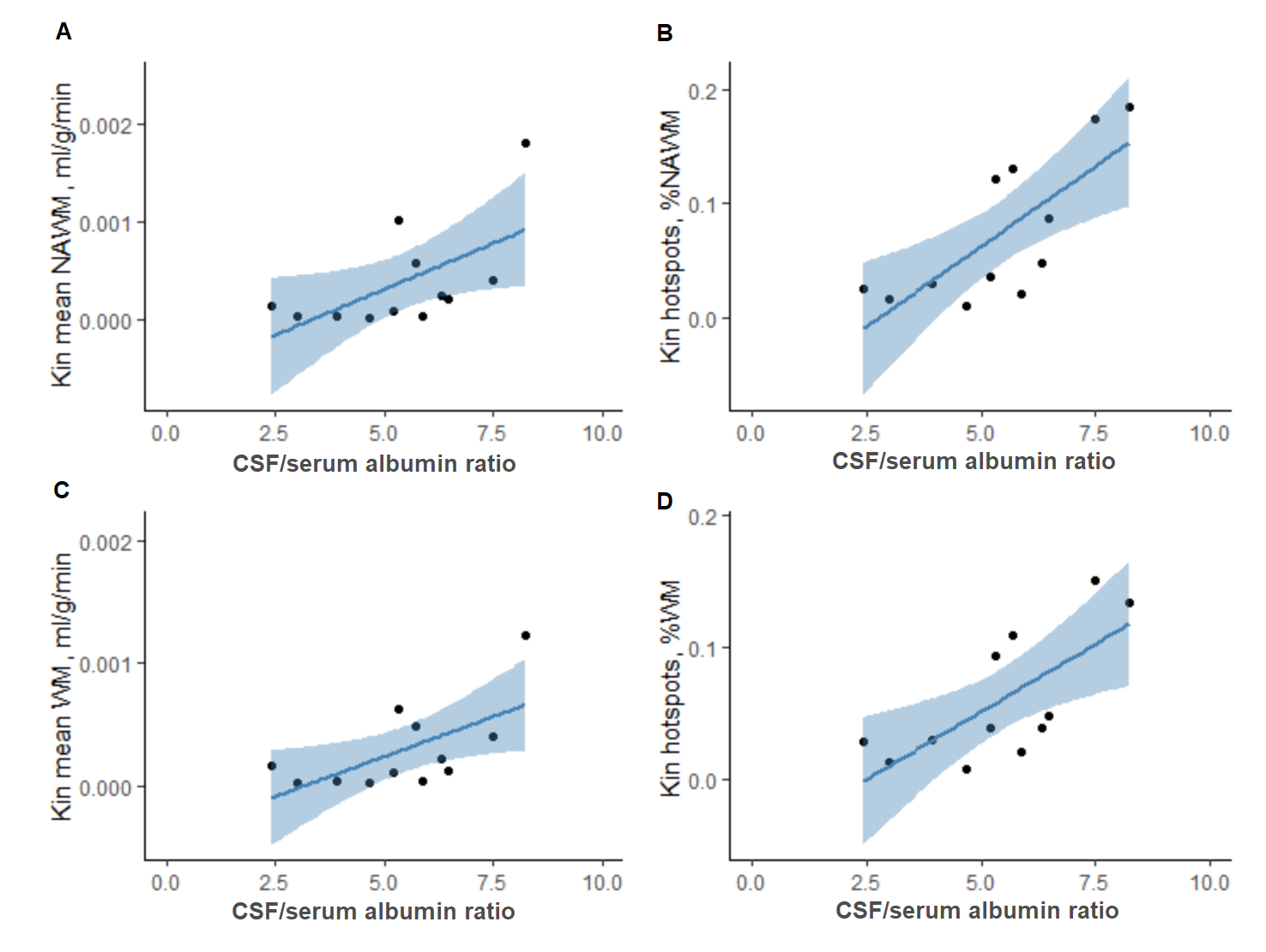
**
